# Personal exposures to traffic-related air pollution in three Canadian bus transit systems: the Urban Transportation Exposure Study

**DOI:** 10.1038/s41370-020-0242-2

**Published:** 2020-07-16

**Authors:** Keith Van Ryswyk, Greg J. Evans, Ryan Kulka, Liu Sun, Kelly Sabaliauskas, Mathieu Rouleau, Angelos T. Anastasopolos, Lance Wallace, Scott Weichenthal

**Affiliations:** 1 Air Health Science Division, Health Canada, Ottawa, ON K1A 0K9 Canada; 2 Department of Chemical Engineering and Applied Chemistry, University of Toronto, Toronto, ON Canada; 3 Santa Rosa, CA 5409 USA; 4 Department of Epidemiology, Biostatistics, and Occupational Health, McGill University, Montreal, QC H3A 1A2 Canada

**Keywords:** Personal exposure, Bus transit, UFP, PM_2.5_, BC, Metals

## Abstract

**Background:**

Exposure to traffic-related air pollution (TRAP) is associated with increased incidence of several cardiopulmonary diseases. The elevated TRAP exposures of commuting environments can result in significant contributions to daily exposures.

**Objectives:**

To assess the personal TRAP exposures (UFPs, BC, PM_2.5_, and PM_10_) of the bus transit systems of Toronto, Ottawa, and Vancouver, Canada. Personal exposure models estimated the contribution of bus commuting to daily TRAP exposures. Associations between bus type and riding exposures and bus stop/station type and waiting exposures were estimated.

**Results:**

Bus commuting (4.6% of the day) contributed ~59%(SD = 15%), 60%(SD = 20%), and 57%(SD = 18%) of daily PM_2.5_-Ba and 70%(SD = 19%), 64%(SD = 15%), and 70%(SD = 15%) of daily PM_2.5_-Fe, in Toronto, Ottawa, and Vancouver, respectively. Enclosed bus stations were found to be hotspots of PM_2.5_ and BC. Buses with diesel particulate filters (DPFs) and hybrid diesel/electric propulsion were found to have significantly lower in-bus PM_2.5_, UFP, and BC relative to 1983–2003 diesel buses in each city with the exception of UFP in Vancouver.

**Significance:**

Personal exposures for traffic-related air pollutants were assessed for three Canadian bus transit systems. In each system, bus commuting was estimated to contribute significantly toward daily exposures of fine-fraction Ba and Fe as well as BC. Exposures while riding were associated with bus type for several pollutants in each city. These associations suggest the use of hybrid diesel/electric buses equipped with diesel particulate filters have improved air quality for riders.

## Introduction

Traffic-related air pollution (TRAP) is ubiquitous in urban environments and constitutes a significant burden on public health. Exposure to TRAP has been found to increase the incidence of several cardiopulmonary diseases [1–4], cancers [5–8], as well as type II diabetes [9], and is related to neurotoxicity [10, 11]. In North America, the transportation sector is estimated to account for 32% of total fine particulate matter (PM_2.5_) mortality [12]. This evidence has led to the adoption of increasingly stringent regulations resulting in reductions of engine and vehicle emissions and regulated air pollutants [13]. In Canada, the reduction of traffic-related PM_2.5_ emissions, as reflected in Canadian air pollutant emission inventories, has likely contributed to a ~25% decrease in Canadian population-weighted mean PM_2.5_ concentration from 2000 to 2011 [14].

Personal exposures to TRAP in commuting environments is a research priority. While commuting represents ~5% of a Canadian’s day [15], this microenvironment can represent a larger proportion of daily exposure to traffic-related air pollutants [16–18]. The characterization of air pollution in urban transport environments can help refine air pollution exposure estimates as well as inform air quality policy. The Urban Transportation Exposure Study (UTES) was designed to assess TRAP exposures in Canadian commuting environments. Results from the subway/metro [18], and private vehicle [19] components of UTES are published elsewhere. This paper presents an air pollution exposure assessment for the public bus transit systems of Toronto, Ottawa, and Vancouver, Canada.

Public bus transit is used by 12.2% of Canadians and their bus commute represents an average of 66 min (4.6%) of their day [20]. While subways are almost exclusively electric propulsion and private vehicles predominantly use gasoline, public bus transit is extensively diesel propulsion. Diesel exhaust is a known source of black carbon (BC) and ultrafine particles (UFPs) and has been designated carcinogenic [21]. Further, BC may play a role in the health effects associated with TRAP [22]. Air pollution exposure data specific to this staple of public transit are valuable for air pollution risk assessment and management.

Several heavy-duty diesel engine exhaust emission standards have been adopted in Canada. For example, emission standards introduced in 2004 targeted the reduction of NO*x* emissions, which were met mainly through the use of exhaust gas recirculation. Exhaust emission standards for diesel engines of model year 2007 and later are ten times lower than previous limits for PM_2.5_ (*On-Road Vehicle and Engine Emission Regulations*, SOR/2003-2). This drastic reduction in the PM emission limit in 2007 coincided with the availability of ultra-low sulfur diesel fuel starting in 2006 (*Sulphur in Diesel Fuel Regulations*, SOR/2002/254) which enabled the use of efficient exhaust after-treatment devices, notably diesel particulate filters (DPFs). these measures to decrease exhaust emissions have reduced ambient air concentrations, their impact on in-bus exposures has not been studied.

Non-exhaust sources of TRAP are a growing concern. A review of the epidemiological evidence of the health effects of non-exhaust particles suggests that it has both short and long term health effects [23]. As well data from long term monitoring at a downtown site in Toronto have indicated that non-exhaust emissions are an increasing component of TRAP [24]. In the context of bus transit environments, they are particularly relevant due to the brake and wheel wear of these heavy vehicles. Sources of non-exhaust emissions include the degradation of roads, brakes, and tires and are typically represented by trace metals [25]. Several trace metals have been associated with increased risk of disease. Exposure to fine-fraction trace elements have been associated with cardiopulmonary hospitalizations and mortality [26–28] and lung cancer [29]. While research on identifying the health burden of fine-fraction trace elements continues, so too should work on estimating the contribution of commuting to the daily exposure of these pollutants. Such evidence is valuable toward informing personal exposure modeling and mitigation.

This work has several research objectives. The first was to characterize the traffic-related air pollutants of nitrogen dioxide (NO_2_), UFPs, BC, PM_2.5_, coarse particulate matter (PM_10_), and the elemental content of PM_2.5_ and PM_10_ for the bus transit systems of Toronto, Ottawa, and Vancouver, Canada. The second was to estimate the contribution of bus commuting to overall daily TRAP exposures. The third was to estimate the potential of bus stations to represent TRAP exposure hotspots by comparing their exposures to that of bus stops. Fourth: the simulation of air quality interventions on the daily means of our waiting and riding exposure data. To this end, our estimates of lower waiting exposures at bus stops (relative to bus stations) were used to estimate the impact of air quality interventions designed to reduce bus station concentrations to that of bus stops. Also, we used our estimates of lower riding exposures in hybrid diesel/electric buses (relative to 1983–2003 buses) to estimate the impact of the replacement of 1983–2003 buses with hybrid diesel/electric buses.

## Methods

### Data collection

**Fig. 1:**
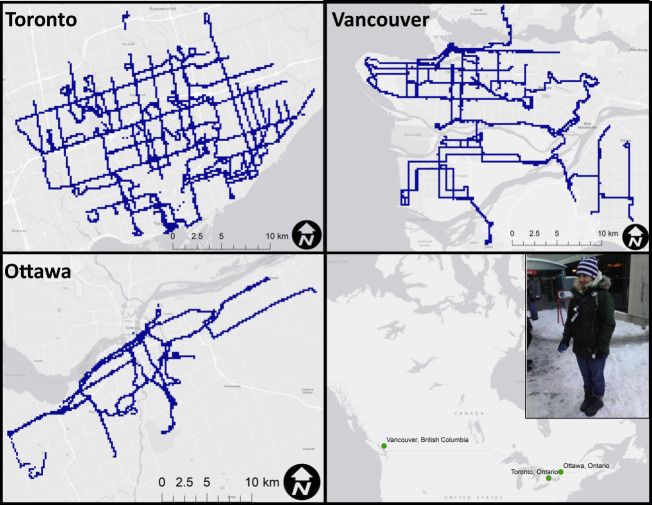
Personal air monitoring set up and geographical coverage of sampled routes in Toronto, Ontario; Ottawa, Ontario; and Vancouver, British Columbia.

Air pollution exposure data were collected in the public bus transit systems of Toronto and Ottawa, Ontario for three weeks in the summer of 2010 and the winter of 2011 and in Vancouver, British Columbia in the winter and summer of 2013. Data were collected during the peak commuting hours of 7–10 a.m. and 3–6 p.m. of each weekday. During each 3-h sampling session, three researchers, assigned to separate areas of the city, carried equipment in personal sampling backpacks (Fig. 1). Bus routes reflecting high ridership and the geographic extent of the network were selected to best reflect exposures for the majority of bus commuters. While sampling each bus route, researchers disembarked at bus stops/stations at regular intervals to ensure the collection of both waiting and riding exposures. This also ensured data collection for a wide variety of bus stop/station types and bus make years and propulsion types. Geographic location data were collected using a global position recorder (GlobalSat DG-100). Digital voice recordings (DVR) were used to identify periods of riding and waiting as well as bus and bus stop IDs.

Continuous data were recorded in 1-s intervals and averaged over each session of waiting and riding. This resulted in a series of data points alternating between waiting and riding over each 3-h period. Sampling inlets for the air pollution monitors were positioned in the breathing zone. Throughout each 3-h monitoring session, PM_2.5_, UFPs, and BC were measured continuously with electronic devices. The internal clocks of all continuous instruments, including GPS data and DVRs, were synchronized to the researcher’s computers on a daily basis. All were merged at 1-s intervals. Continuous data were then averaged over each waiting and riding session. Further detail on the continuous air pollution monitoring instruments and QAQC are provided in Supplementary section 1.1. Filter-based PM_2.5_ and PM_10_ samples were collected concurrently with the continuous data. Each sample represented the 30 h of sampling conducted by a researcher each week for a total of 18 PM_2.5_ and PM_10_ samples for each city. Further detail on the integrated PM_2.5_ and PM_10_ methods can be found in Supplementary section 1.2.

### Estimating the contribution of bus commuting to daily exposures

Microenvironmental models combining bus and ambient exposure data were created to estimate the contribution of bus commuting to overall daily exposures of PM_2.5_ and PM_2.5_-elements as well as BC and UFP. Since these models pertain to the impact of commuting, their context applies only to weekdays. The microenvironmental model for PM_2.5_ and fine-fraction elements combined the personal bus commuting exposure data collected in this study with fixed-site ambient monitoring data and a correction factor to account for the differences in PM_2.5_ sampling methods. The microenvironmental model for BC and UFP differed from the PM_2.5_ model. With the high temporal resolution of the BC and UFP data collected in this study, exposures specific to riding and waiting were calculated. Also, in place of available ambient data for UFP and BC, estimates were made for three (“low”, “moderate”, and “high”) ambient conditions. Both approaches used the figure of 66 min as the amount of time spent in the bus environment [20].

For estimating the percent contribution of bus commuting to weekday PM_2.5_ and PM_2.5_-elemental exposures, ambient (non-commuting) exposures were represented by data downloaded from the National Air Pollution Surveillance (NAPS) network operated by Environment and Climate Change Canada (ECCC). ECCC conducts the routine collection of 24-h gravimetric PM_2.5_ for several stations across Canada every 3 days. One NAPS site in each city (Toronto, Ottawa, and Vancouver) collected gravimetric data for the study period. NAPS data collected during and within 2 weeks of each six sampling sessions (three cities × two seasonal sampling periods) were downloaded to represent ambient levels of PM_2.5_ and its elemental composition. These data were then averaged to produce mean levels for each city and season. Elements which were measured to be above detection for >50% of samples in both the bus sampling and ambient environments were included in the analysis along with PM_2.5_ itself. Bus commuting exposures were represented by the 18 integrated PM_2.5_ samples collected in each city (Supplementary section 1.2). The percent contribution of bus commuting to overall PM_2.5_ components (mass and elemental concentration) was calculated with each bus sample and its corresponding ambient seasonal mean (Eq. 1). G_busPM2.5*ijk*_ represents the estimated contribution of a typical 66-min bus commute to PM_2.5_ exposure for sample *i*, PM_2.5_ component *j*, and season *k*. *X*_busPM2.5*ij*_ represents the concentration of PM_2.5_ component *j* for sample *i*, *X*_AmbientPM2.5jk_ is the mean concentration of PM_2.5_ component *j* for season *k*, and *A*_PEM:Dichot_ represents the correction factor for the 11% positive bias of the PEM, relative to the dichotomous sampler used by NAPS [30]. The mean and standard deviation of the resulting values (*n* = 18) were then calculated for each city.

1 $${\it{G}}_{bus\,PM_{2.5\,ijk}} = \\ 	 {\frac{{{Bus\,PM_{2.5\,ij} \, \times\, A_{PEM:Dichot} \,\times \,66\,mins}}}{[ {Bus\,PM_{2.5\,ij}\, \times \,A_{PEM{\mathrm{:}}Dichot}\, \times \,66\,mins} ] + [ {\overline X \,Ambient\,PM_{2.5\,jk}\, \times \,1374\,mins} ]}}$$

The percent contribution of bus commuting to overall daily exposures of BC and UFP was estimated using the continuously monitored BC and UFP data. As with the PM_2.5_ analysis, the figure of 66 min was used as the time spent on buses, however, a weighted average between time spent waiting at bus stations (16 min—based on mean waiting time (Supplementary Table S2)) and time spent on buses (50 min) was calculated to represent bus exposure. Ambient data from NAPS for BC and UFP were largely unavailable for these cities during these time periods. Therefore, the percent contribution of bus commuting for overall daily BC and UFP exposure was calculated for three scenarios of mean ambient (non-commuting) exposure. For BC, these were 0.5, 1.0, and 1.5 µg/m^3^ and 5, 10, and 15 × 10^3^ pts/cm^3^ for UFP. These values of “low”, “moderate”, and “high” non-commuting exposures were selected to represent the variation possible in consideration of how much time a person spends indoors and in close proximity to busy roads. The microenvironmental model for estimating the percent contribution of bus commuting to daily exposure to UFP and BC is presented in Eq. (2) where *G*_bus ijk_ represents the estimated contribution of a typical 66-min bus commute for sampling day *i*, pollutant *j*, and ambient condition *k*. *X*_waiting ij_ represents the mean bus stop (waiting) exposure for day *i* and pollutant *j*, *X*_riding ij_ represents the mean in-bus (riding) exposure for day *i* and pollutant *j*, and Ambient_j*k*_ represents the mean pollutant *j* for ambient scenario *k* (“low” “moderate”, or “high”). The mean and standard deviation of the resulting values were then calculated for each city (*n* = 30).

2 $$G_{{bus}_{ijk}} = \\ 	 \frac{{[ {\overline X _{waiting_{ij}} \,\times \, 16\,min} s]\, +\, [ {\overline X _{riding_{ij}}\, \times \, 50\,mins} ]}}{[ {\overline X _{waiting_{ij}}\, \times\, 16\,min} s]\, +\, [ {\overline X _{riding_{ij}}\, \times\,50\,min} s] \,+\, [ {\overline X \,Ambient_{jk} \,\times\, 1374\,mins} ]}$$

### Impact of bus stop type and bus type on PM_2.5_, UFP, and BC exposures

Upon boarding a bus or disembarking at a stop, DVR recordings were made of each bus and stop ID. Bus IDs were cross-referenced with transit authority fleet data on manufacture year and propulsion type. This information was used to classify each bus type as 1983–2003 diesel, 2004–2006 diesel, 2007- diesel, hybrid diesel/electric, or electric. This classification scheme reflects the adoption of heavy-duty diesel engine exhaust emission standards in Canada, which have generally mirrored those from the United States. The 1983–2003 diesel bus category includes older technology engines that meet less stringent emission standards. The 2004–2006 category represents buses designed to meet 2004 emission standards. The post 2007 category represents buses, which feature DPFs. The hybrid diesel/electric propulsion systems are based on different engine characteristics, operating regimes, and fuel consumption profiles compared with conventional diesel engines. They are considered a distinct class, irrespective of model year. Electric buses are also a distinct class, irrespective of model year, as they do not emit exhaust emissions. Bus stops were classed as “bus stop” (street level outdoor stop), “outdoor bus station” (street level outdoor station serving as a hub for several bus routes), and “enclosed bus station” (indoor/below grade station serving as a hub for several bus routes). GPS data of “waiting” sessions were plotted in ArcGIS to confirm bus stop classifications.

Multivariate linear mixed models were developed to estimate differences in exposure between each class of bus stop and bus type and a referent class. As these conditions changed throughout each 3-h monitoring period, this analysis was only possible with continuously monitored pollutants (PM_2.5_, UFP, and BC). Waiting exposure data were used to test for a significant difference between exposures at bus stations (outdoor and enclosed) and bus stops (the referent class). Riding exposure data were used to test for a significant difference between the exposures of the various bus types (2004–2006 diesels, 2007- diesels, hybrid diesel/electric, and electric) and 1983–2003 diesels (the referent class). In total, nine models were developed to estimate the effect of bus stop and bus type (one for each combination of city and continuously monitored pollutant). The multivariate mixed-effects linear regression models included adjustment for confounders in order to isolate the effect of the main predictor (bus stop type and bus type). A directed acyclic graph (DAG) was used to inform the selection of confounding variables. Specifically, we identified the minimal sufficient adjustment set of variables for estimating the effect of bus type on riding exposures and bus stop type on waiting exposures. Several types of land use (commercial, industrial, residential, open space, and parks) and road types (local roads, major roads, and highways) were identified as potential confounders (Supplementary Fig. S2). Controlling for factors of land-use and road network information was essential as they can affect air pollutants in bus transit [31, 32]. Each confounder was represented as the quantity within a 500 m buffer. The DAG was built using DAGitty version 3.0 (http://www.dagitty.net/). Estimates of the relationship between bus type and bus stop type and exposure levels were calculated using the “proc mixed” procedure in SAS EG 5.1 (SAS Institute Inc., Cary, NC, USA). An autoregressive covariance structure (AR_1_) was used to account for the lack of independence within the repeated measurements within each 3-h session (Eq. 3).

3 $$Y_{ij} = \alpha + \beta _1X_1 + \beta _2X_2 + \ldots + \beta _nX_n + b_i + \varepsilon _{ij}$$

Here, *Y*_*ij*_ is the natural logarithm for PM_2.5_, UFP, or BC for riding/waiting session *i*, on day *j*, α is the regression intercept, *β*_1_ is the effect of bus stop/bus type (*X*_1_) on Y, *β*_2_ is the effect of the first confounder of *n* confounders, *b*_*i*_ is the random effect for propulsion/stop type and *ε*_*ij*_ is the random error. Effects of influential points were assessed by calculating Cook’s distances. Residual plots were assessed for normality using the Shapiro-Wilk statistic. Predictor parameter estimates and 95% confidence intervals were exponentiated to provide estimates of percent change in exposure. Significant differences between outdoor and enclosed bus stations and bus stops were expressed as percent increases in exposure. Significant differences in exposures between the 2004–2006 diesels, 2007- diesels, hybrid diesel/electric, and electric bus types and the referent 1983–2003 bus type were expressed as percent decreases.

### Estimating the impact of simulated bus fleet renewal and bus station air quality interventions

Results of the mixed model analyses were applied to simulate the impact of interventions designed to improve waiting and riding exposures of PM_2.5_, UFP, and BC. This analysis combined the effect size of lower waiting concentrations at bus stops and riding concentrations in hybrid diesel/electric buses with the frequency in which they were encountered during sampling. For waiting exposures, mixed model estimates of higher exposures for outdoor and enclosed bus stations (relative to bus stops) were used to estimate the impact of improving bus station air quality. For riding exposures, mixed model estimates of lower exposures for hybrid diesel/electric buses (relative to the 1983–2003 class) were used to estimate the impact of bus fleet changeover (replacement of 1983–2003 buses with diesel hybrid/electric). Daily means of waiting and riding concentrations were calculated before and after the application of air quality improvements. Percent reduction of waiting and riding exposures were then calculated for each daily mean. The mean and standard deviation of the resulting values (*n* = 30) were then presented for each.

## Results

### Data collection and TRAP exposure levels

Technicians collected exposure data for ~900 (Toronto), 1700 (Ottawa), and 1400 (Vancouver) riding and waiting sessions (Supplementary Table S2). Riding sessions lasted an average of 17, 8, and 14 min in Toronto, Ottawa, and Vancouver, respectively. Waiting sessions were on average ~8 min in all three cities. A description of TRAP exposure levels with breakdowns by city and season (Supplementary Table S3), waiting/riding (Supplementary Table S4), bus type (Supplementary Table S5), and bus stop type (Supplementary Table S6) can be found in section 2 of the Supplementary. Results of the PM_2.5_ and PM_10_ integrated samples are presented in Supplementary Table S7.

### Contribution of bus commuting to daily exposure of PM_2.5_ mass, its elemental constituents, BC, and UFP

The gravimetric PM_2.5_ bus commuting exposures were combined with central site ambient data using a personal exposure model to estimate the percent contribution of bus commuting towards mean daily exposures. A total of 21 central site ambient samples were available in each city and 18, 17, and 14 elements met the inclusion criteria for this analysis for Toronto, Ottawa, and Vancouver, respectively. Descriptive statistics for bus and ambient exposure for PM_2.5_ and these elemental constituents are presented in Supplementary Tables S8A (Toronto), S8B (Ottawa), and S8C (Vancouver). A summary of the percent contribution estimates are presented in Table 1. A typical 66 min bus commute (4.6% of the day) was estimated to contribute 6% (SD = 2%) (Toronto), 13% (SD = 10%) (Ottawa), and 11% (SD = 4%) (Vancouver) of weekday PM_2.5_ exposure. Daily exposure to several elements were highly influenced by bus commuting. Time spent bus commuting was estimated to contribute 59% (SD = 15%) (Toronto), 60% (SD = 20%) (Ottawa), and 57% (SD = 18%) (Vancouver) of daily exposure to fine-fraction Ba, and 70%(SD = 19%) (Toronto), 64% (SD = 15%) (Ottawa), and 70% (SD = 15%) (Vancouver) of daily exposure to fine-fraction Fe. Contributions of over 25% in each city were also noted for Al, Cu, and Ti.

**Table 1:** Contribution of bus commute to daily exposure of PM_2.5_ mass and elements.

Element	Mean (SD) % contribution^a^		
	Toronto (*n* = 18)	Ottawa (*n* = 18)	Vancouver (*n* = 17)
PM_2.5_	6 (2)	13 (10)	11 (4)
Al	26 (11)	32 (23)	38 (15)
As	7 (3)	5 (3)	8 (2)
Ba	59 (15)	60 (20)	57 (18)
Cd	8 (7)	10 (9)	–
Cr	27 (21)	19 (18)	–
Co	–	–	21 (9)
Cu	30 (15)	37 (9)	32 (7)
Fe	70 (19)	64 (15)	70 (15)
Mn	27 (14)	23 (13)	18 (9)
Mo	17 (12)	24 (7)	22 (6)
Ni	15 (11)	22 (16)	14 (9)
Pb	12 (6)	10 (5)	18 (13)
Sb	12 (4)	10 (6)	36 (10)
Se	4 (2)	–	–
Sn	17 (4)	19 (8)	–
Sr	20 (9)	26 (15)	24 (9)
Ti	39 (14)	41 (19)	–
V	6 (2)	8 (5)	13 (6)
Zn	10 (9)	12 (11)	8 (2)

Full table can be found in Supplementary (S8A, B, C).

^a^Percent contribution of 66-min bus commute (4.6% of day) to overall daily exposure (Eq. 1).

Estimates of the contribution of bus commuting to daily BC and UFP exposure are presented in Table 2. Bus commuting contributed 7.1–8.9% of overall daily UFP exposures in the “moderate” ambient (non-commuting exposure) condition of 5 × 10^3^ pts/cm^3^. For BC, bus commuting contributed 19.1–23.0% of daily BC exposures in the “low” ambient (non-commuting) exposure scenario of 0.5 µg/m^3^.

**Table 2:** Percent contribution of bus commute to daily UFP and BC for a range of ambient levels.

Pollutant	City	Mean daily concentration (SD)		Mean % contribution by ambient condition (SD)^a^		
		Riding	Waiting	Low	Moderate	High
Ultrafine	Toronto	16.2 (4.1)	24.5 (7.5)	14.7 (3.3)	8.0 (1.9)	5.5 (1.3)
particles	Ottawa	18.9 (8.5)	26.9 (15.5)	16.2 (6.4)	8.9 (3.9)	6.2 (2.8)
(10^3^ pts/cm^3^)	Vancouver	16.1 (12.1)	18.4 (14.2)	13.0 (8.6)	7.1 (5.0)	4.9 (3.5)
Black	Toronto	3.1 (2.0)	4.6 (4.3)	23.0 (11.9)	13.5 (8.0)	9.6 (6.0)
Carbon	Ottawa	2.6 (1.4)	2.8 (1.5)	19.4 (9.0)	11.0 (5.5)	7.7 (4.0)
(µg/m^3^)	Vancouver	2.6 (0.6)	2.0 (0.6)	19.1 (3.5)	10.6 (2.1)	7.3 (1.5)

Calculated for each daily mean waiting and riding (*n* = 30). Low ambient: 5 × 10^3^ pts/cm^3^ UFP and 0.5 µg/m^3^ BC; moderate ambient: 10 × 10^3^ pts/cm^3^ UFP and 1.0 µg/m^3^ BC; high ambient: 15 × 103 pts/cm^3^ UFP and 1.5 µg/m^3^ BC.

^a^Percent contribution of 66-min bus commute (4.6% of day) to overall daily exposure (Eq. 2).

### Effect of bus stop type on waiting exposures of PM_2.5_, UFP, and BC

The % increases in PM_2.5_, UFP, and BC exposure for outdoor and enclosed bus stations, relative to bus stops, are presented in Table 3. In Toronto, PM_2.5_ levels for outdoor bus stations were 55% (95% CI: 39–73%) higher than bus stops while enclosed bus stations were 79% (95% CI: 61–100%) higher. UFP levels were slightly higher for outdoor bus stations in Ottawa (25% 95% CI: 15–37%) relative to bus stops but this difference was not seen for enclosed bus stations. In Toronto, UFP levels were counterintuitively lower at bus stations relative to bus stops, both outdoor and enclosed. In Toronto and Ottawa, increases in BC levels were noted at outdoor bus stations with further increases at enclosed bus stations. BC exposures at outdoor bus stations were significantly higher than bus stops in Toronto (64%, 95% CI: 30–107%) and Ottawa (27% 95% CI: 14–42%). As well, enclosed bus stations showed further increases in BC levels as they were 112% (95% CI: 66–172%) higher than bus stops in Toronto and 56% (95% CI: 22–99%) higher in Ottawa. No enclosed bus stations were encountered in Vancouver and only a small sample of bus station data were collected.

**Table 3:** Percent increase of exposures at outdoor and enclosed bus stations, relative to exposures at bus stops.

Pollutant	Bus stop type	Toronto			Ottawa			Vancouver^a^		
		*n*	% increase	95% Cl (LCL, UCL)	*n*	% increase	95% Cl (LCL, UCL)	*n*	% increase	95% Cl (LCL, UCL)
PM_2.5_	Outdoor bus station	114	55	39, 73	768	ns	ns	38	ns	ns
	Enclosed bus station	101	79	61, 100	50	15	3, 30	0	–	–
	Bus stop^b^	623			592			1362		
Ultrafine particles	Outdoor bus station	85	−22	−31, −11	731	25	15, 37	37	ns	ns
	Enclosed bus station	91	−14	−23, −3	47	ns	ns	0	–	–
	Bus stop^b^	478			555			1314		
Black Carbon	Outdoor bus station	102	64	30, 107	757	27	14, 42	35	ns	ns
	Enclosed bus station	87	112	66, 172	51	56	22, 99	0	–	–
	Bus stop^b^	504			576			1177		

“ns” denotes a nonsignificant increase in exposure, relative to the referent condition.

^a^No enclosed bus stations were sampled in Vancouver.

^b^Referent condition.

### Effect of bus type on riding exposures of PM_2.5_, UFP, and BC

Percent decreases in PM_2.5_, UFP, and BC exposures by bus type relative to the referent condition (1983–2003 diesel) are presented in Table 4. Across the nine groups defined by TRAP pollutant and city, both the ‘hybrid electric/diesel’ and ‘2007- diesel’ classes were found to have the most cases of % decreases in exposure relative to the “1983–2003” class. Exposures on hybrid electric/diesel buses were significantly lower than the referent group in PM_2.5_, UFP, and BC in all three cities with the exception of UFPs in Vancouver. The 2007- diesel buses were associated with lower PM_2.5_ in Toronto (38%, 95% CI; 29–46%) and Vancouver (40%, 95% CI; 36–45%), and lower UFP in Toronto (40%, 95% CI; 27–50%). The 2007- diesel buses also had significantly lower BC levels in all three cities (Toronto: 38%, 95% CI; 23–50%), Ottawa: (24%, 95% CI; 16–32%), and Vancouver: (20%, 95% CI; 11–29%)). The 2004–2006 diesel buses were estimated to have lower PM_2.5_ in Vancouver (18%, 95% CI; 9–27%), lower UFP in Toronto (30%, 95% CI; 17–40%) and counterintuitively higher UFP in Vancouver (−22%, 95% CI; −42 to −5%).

**Table 4:** Percent decrease of in-bus exposures by bus types, relative to then 1983–2003 diesel bus class.

Pollutant	Bus type	Toronto			Ottawa			Vancouver		
		*n*	% decrease	95% CL (LCL, UCL)	*n*	% decrease	95% CL (LCL, UCL)	*n*	% decrease	95% CL (LCL, UCL)
PM_2.5_	Electric^a^	0	–	–	0	–	–	313	40	33, 46
	Hybrid diesel/electric	407	31	22, 38	242	25	15, 34	219	44	39, 49
	Diesel 2007-	107	38	29, 46	598	ns	ns	366	40	36, 45
	Diesel 2004–2006	269	ns	ns	276	ns	ns	57	18	9, 27
	Diesel 1983–2003^b^	147			415			405		
Ultrafine particles	Electric^a^	0	–	–	0	–	–	307	ns	ns
	Hybrid diesel/electric	310	23	10, 34	218	36	24, 47	213	ns	ns
	Diesel 2007-	89	40	27, 50	586	ns	ns	350	ns	ns
	Diesel 2004–2006	243	30	17, 40	241	ns	ns	54	−22	−42, −5
	Diesel 1983–2003^b^	79			392			386		
Black Carbon	Electric^a^	0	–	–	0	–	–	269	30	19, 40
	Hybrid diesel/electric	322	20	5, 32	243	43	33, 51	183	25	15, 34
	Diesel 2007-	102	38	23, 50	581	24	16, 32	308	20	11, 29
	Diesel 2004–2006	232	ns	ns	275	ns	ns	56	ns	ns
	Diesel 1983–2003^b^	146			398			388		

“ns” denotes a nonsignificant increase in exposure, relative to the referent condition.

^a^Electric buses were unique to Vancouver.

^b^Referent condition.

### Estimated impact of bus station air quality interventions

As per the results presented in Table 3, both outdoor and enclosed bus stations in Toronto had significantly higher PM_2.5_ and BC exposures. In Ottawa, outdoor and enclosed bus stations had significantly higher BC exposures while only outdoor bus stations had higher UFPs and enclosed stations had higher PM_2.5_. The counterintuitively lower UFP exposures at Toronto bus stations were not applied in this analysis. The impact of the simulated reduction of bus station exposures to that of bus stops on the mean daily waiting exposures measured in this study are presented in Table 5. In Toronto, overall waiting PM_2.5_ exposures were reduced by 13% (SD = 7.5%) and BC was reduced by 11.4%(SD = 2.6%). In Ottawa, waiting exposures were reduced for PM_2.5_ (mean = 0.5%, SD = 0.85%), UFP (mean = 11.4%, SD = 2.6%), and BC (mean = 14.1%, SD = 4.1%).

**Table 5:** Estimated reductions to mean waiting exposures with the simulation of station exposures being reduced to that of bus stops.

Pollutant	City	Mean daily concentration (SD)		Mean % reduction of waiting exposures (SD)
		Sampled data	After the reduction of bus station exposures to that of bus stops^a^	
PM_2.5_ (µg/m^3^)	Toronto	27.5 (14.2)	24.3 (13.6)	13 (7.5)
	Ottawa	24.2 (14.4)	24.1 (14.3)	0.5 (0.8)
	Vancouver	15.2 (5.6)	–	–
Ultrafine particles (10^3^ pts/cm^3^)	Toronto	23.5 (8.1)	–	–
	Ottawa	26.8 (15.7)	23.6 (13.6)	11.4 (2.6)
	Vancouver	18.5 (14.1)	–	–
BC (µg/m^3^)	Toronto	4.5 (4.3)	4.1 (4.3)	15.8 (13.2)
	Ottawa	2.9 (1.6)	2.5 (1.3)	14.1 (4.1)
	Vancouver	1.9 (0.6)	–	–

“–” denotes no evidence of higher stations exposures, relative to bus stops.

^a^Based on the percent increases of exposure for at outdoor and enclosed bus stations, relative to bus stops (Table 3).

### Estimated impact of bus fleet renewal

The simulated impact on the daily mean riding exposures measured in this study by the replacement of 1983–2003 buses with hybrid diesel/electric buses is presented in Table 6. The hybrid diesel/electric class was used for this simulation as it was found to have significantly lower exposures in eight of the nine combinations of city and pollutant. In our sample, the 1983–2003 bus class was encountered 15% (Toronto), 27% (Ottawa), and 39% (Vancouver) of the time researchers boarded a bus. Riding exposures were reduced by an average of 6.3% (SD = 5.2%), 6.6% (SD = 4.8%), and 17.6% (SD = 7.5%) for PM_2.5_ and 4.6% (SD = 4.0%), 12.7% (SD = 8.9%), and 9.9% (SD = 4.1%) for BC in Toronto, Ottawa, and Vancouver, respectively. Mean percent reductions for riding UFP exposures were 3.6% (SD = 3.1%) in Toronto and 10.7% (SD = 7.6%) in Ottawa.

**Table 6:** Estimated reduction of overall bus riding exposures with replacement of 1983–2003 buses with hybrid diesel/electric buses.

Pollutant	City	Bus riding exposures (mean (SD))		Estimated % reduction of riding exposures with fleet changeover
		Sampled fleet	With replacement of 1983–2003 buses	
PM_2.5_ (µg/m^3^)	Toronto	22.5 (9.9)	21.3 (9.9)	6.3 (5.2)
	Ottawa	24.2 (12.6)	22.6 (11.4)	6.6 (4.8)
	Vancouver	18.2 (5.5)	15.0 (4.8)	17.6 (7.5)
Ultrafine particles (10^3^ pts/cm^3^)	Toronto	15.8 (4.4)	15.2 (4.3)	3.6 (3.1)
	Ottawa	18.3 (8.6)	16.7 (8.6)	10.7 (7.6)
	Vancouver	16 (12.0)	–	–
BC (µg/m^3^)	Toronto	3.1 (2.0)	3.0 (1.9)	4.6 (4.0)
	Ottawa	2.6 (1.5)	2.1 (1.1)	12.7 (8.9)
	Vancouver	2.6 (0.6)	2.4 (0.5)	9.9 (4.1)

“–” denotes no evidence of lower UFP exposures in diesel/electric hybrids relative to the 1983–2003 class.

^a^Based on estimates of % reduced exposure presented in Table 4.

## Discussion

### Contribution of bus commuting to daily TRAP exposures

A range of fine-fraction elemental bus exposures were noted to be higher than ambient in all three cities, in particular Al, Ba, Cu, Fe, Mn, and Ti. These elements are known to be related to non-exhaust emissions [24]. The concentrations of these elements were at least an order of magnitude greater in the bus environment relative to ambient. This resulted in the 66 minute bus commute (4.6% of the day) contributing the majority of daily exposures to these elemental concentrations. This was particularly the case for Fe and Ba where over 50% of daily exposure was estimated to originate from bus commuting in all three cities. These elements are expected to originate from brake wear as iron oxides and barium can constitute a large proportion of brake lining material [33]. The elemental composition of in-bus PM_2.5_ has also been studied in Barcelona, Spain where 48 samples were collected, each representing three 2-h monitoring sessions [34]. Elemental markers of brake dust were also found to be significantly higher than urban background. In contrast to this study, brake wear was represented by the elements Sb and Cu. Fine-fraction concentrations of Sb and Cu were lower for routes with few gradients relative to routes with many. They were also notably lower in electrical buses relative to diesels which the authors attributed to the presence of regenerative braking in electric buses.

For the estimates of the contribution of bus commuting to daily UFP and BC exposure, commuting exposure was represented as a time-weighted average of waiting and riding exposures. This was an important measure on account of the significant difference of exposures noted between these two environments (Supplementary Table S4). In all three cities, UFP was higher in the waiting mode and BC levels were significantly different between the waiting and riding modes in Toronto and Vancouver. Bus commuting was estimated to constitute a fifth of daily BC exposure in each city in the ‘low’ ambient (non-commuting) scenario of 0.5 µg/m^3^. These estimates were lower than other studies which measured personal exposures throughout the day. In Australia, personal BC exposures were measured for one individual for twenty 24-h periods. Bus commuting was estimated to contribute 36% of daily exposure [35]. As well, the personal BC monitoring of 26 individuals for 7 days estimated time spent in the “transport” activity to contribute 25.6% of daily BC exposure [36], however, the ‘transport’ activity included a variety of methods. Bus commuting was estimated to contribute less of daily UFP than BC. In the ‘moderate’ ambient (non-commuting) scenario of 10 × 10^3^ pts/cm^3^, bus commuting contributed a mean of 7.1–8.9% of daily UFP exposure across the three cities. In two Canadian studies, mean indoor and outdoor UFP concentrations of 10 × 10^3^ pts/cm^3^ were common. A study of 50 homes in Windsor found mean indoor levels of 10 (SD = 27) × 10^3 ^pts/cm^3^ in winter and 8 (SD = 20) × 10^3^ pts/cm^3^ in summer [37]. In Edmonton, 50 homes were also studied for one week in summer and winter where indoor UFPs were 10 (SD = 25) in winter and 9 (SD = 19) in summer [38]. Mean outdoor UFPs in both of these studies were also within 5–10 × 10^3^ pts/cm^3^. The wide range of indoor and outdoor UFPs across homes measured in both of these studies suggest that there is considerable variation in the contribution of bus commuting to daily UFP, which is not reflected in our estimates.

### Bus stations as hotspots of exposure

**Fig. 2:**
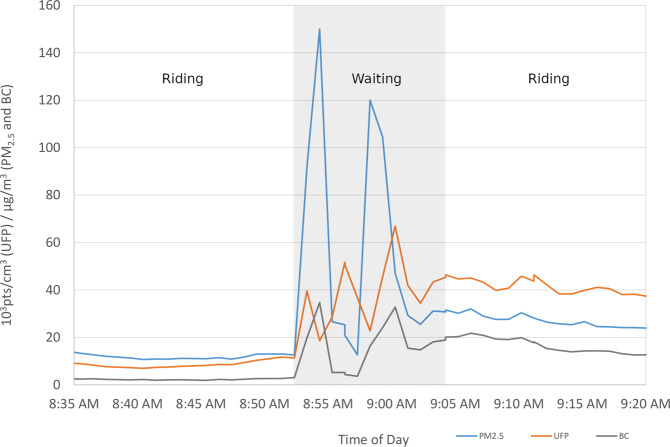
An example of the impact of elevated bus station exposures on riding exposures: a time series plot displaying the minutely PM_2.5_, UFPs, and BC concentrations of a bus ride to an enclosed bus station, a 12 minute waiting period at the enclosed bus station, and the subsequent ride.

Bus stations in Toronto and Ottawa were estimated to have elevated concentrations of BC and PM_2.5_, relative to bus stops. Further, several bus stations were shown to be hotspots of BC exposures (Supplementary Figs. S9–S11) some of which exceeded a recent proposed 8-h occupational standard for elemental carbon (5 µg/m^3^) [39]. There was also evidence that elevated bus station levels could influence in-bus levels. In one example, the PM_2.5_, UFP, and BC concentrations of an enclosed bus station persisted in a bus for up to 20 min after it had left the station (Fig. 2). These in-bus exposures were also substantially higher than the ride that preceded it. This points to the potential for bus station air quality improvements to provide benefits to riding exposures as well.

### Impact of bus propulsion emission reduction systems on riding exposures

Relative to the 1983–2003 diesel, the “2007- diesel” class featured lower in-bus BC exposures in all three systems as well as PM_2.5_ in Toronto and Vancouver and UFPs in Toronto. These lower PM_2.5_, UFP and BC levels are likely due to the use of DPFs. DPFs have been installed on most new heavy-duty diesel vehicles and buses since 2007 in order to comply with PM_2.5_ exhaust emission standards. Similarly, selective catalytic reduction (SCR) systems have been used since 2010 to meet the more stringent NO*x* exhaust emissions standards. DPFs have a significant effect on PM mass, especially the EC/BC/soot fractions compared to engines without them [40]. As buses in the “hybrid diesel/electric” class were all post-2006 make years, they also meet the same vehicle emissions standards as the “2007- diesel” class. Therefore, a comparison of the percent decreases in TRAP exposures between these two bus classes provides an indication of the potential for the hybrid diesel/electric technology to reduce in-bus exposures. The results of these comparisons revealed the same trend for each pollutant within each city, but differed between cities. In Ottawa, the exposure reductions for hybrid buses surpassed that of the “2007- diesel” class for each pollutant. In Vancouver, both of these bus types are similar in their percent reductions while in Toronto, the “2007- diesel’ class” reductions surpassed that of the hybrid diesel class. Similar work examining the impact of bus fleet emission standards on in-bus TRAP exposures was also conducted in the bus rapid transit system of Bogotà, Columbia [41]. This research featured monitoring over 10 months of a year and covered a large proportion of the bus transit network. Emission standards were also found to impact in-bus exposures. Buses with Euro II–III nominal emission standards were found to have significantly higher levels of PM_2.5_ and BC relative to Euro IV–V buses. However, the emissions standards of this bus fleet were older than those in our study. The Euro IV–V class roughly corresponds to post-2005 make years. This difference in the emission standards of this bus fleet was also reflected in significantly higher in-bus levels of PM_2.5_ (mean(SD) = 176.3(137.8) µg/m^3^) and BC (mean(SD) = 89.9(85.8) µg/m^3^).

### Estimated impact of bus station air quality interventions

Exposures at bus stations were associated with higher levels than bus stops, mainly for PM_2.5_ and BC in Toronto and Ottawa. These associations were used to estimate the impact of air quality interventions at bus stations. We also found evidence that hybrid diesel/electric buses provided better air quality for their riders relative to 1983–2003 diesel buses. This finding was consistent for each city and continuously monitored pollutant (PM_2.5_, UFP, and BC) with the exception of UFPs in Vancouver. This suggests the potential for air quality improvements for bus riders with the replacement of 1983–2003 buses with that of hybrid diesel/electric buses, and the reduction of bus station levels to that of bus stops. This potential was explored by estimating the impact of such changes on the exposures measured in this study. This analysis combined the effect size of these associations and the frequency with which the 1983–2003 buses and bus stations were encountered during sampling. The impact of bus fleet turnover was more extensive than reducing bus station exposures due to the magnitude of effect, consistency of this finding across cities and pollutants, and the frequency of encountering 1983–2003 buses. It is also likely that continued bus fleet renewal will contribute to better air quality in waiting environments as well.

### Comparison with UTES private vehicle and subway concentrations

The UTES was designed to assess TRAP exposures in Canadian subway [18], private vehicle [19], and bus commuting environments. The predominant use of diesel propulsion in buses may result in higher exposure to diesel exhaust in buses, relative to private vehicles and subways. UTES investigated all three modes in both Toronto and Vancouver. In Toronto, subway UFPs were lowest (p50(IQR) = 9.1(6.2–12.4) × 10^3^ pts/cm^3^) where bus UFPs (summer p50(IQR) = 15.0(8.6–24.2) × 10^3^ pts/cm^3^; winter p50(IQR) = 17.3(10.7–27.7) × 10^3^ pts/cm^3^) were higher. However, private vehicle UFPs were highest (summer p50(IQR) = 28.6(14.3–54.8) × 10^3^ pts/cm^3^; winter p50(IQR) = 25.5(9.5–133.6) × 10^3^pts/cm^3^). In Vancouver, this comparison of UFP levels revealed the same trend. Subway UFP were lowest (p50(IQR) = 7.0(3.8–18.3) × 10^3^ pts/cm^3^), followed by bus (summer p50(IQR) = 4.8(3.0–7.5) × 10^3^ pts/cm^3^; winter p50(IQR) = 20.4(13.8–38.6) × 10^3^ pts/cm^3^) and then private vehicle (summer p50(IQR) = 20.6(2.8–57.9) × 10^3^ pts/cm^3^; winter p50(IQR) = 30.9(9.6–66.8) × 10^3^ pts/cm^3^). For BC, the comparison of bus levels with that of the subway is not possible on account of subway BC measurements being highly influenced by iron oxides which can be prevalent in subway systems [42]. Toronto bus transit BC (summer p50(IQR) = 3.2(1.7–5.9) µg/m^3^; winter p50(IQR) = 1.1(0.6–2.1) µg/m^3^) was similar to that of private vehicle (summer p50(IQR) = 1.9(0.3–6.9) µg/m^3^; winter p50(IQR) = 1.1(0.5–1.9) µg/m^3^). In Vancouver, the same trend was noted. Bus transit BC (summer p50(IQR) = 1.7(1.0–2.9) µg/m^3^; winter p50(IQR) = 1.8(1.1–3.1) µg/m^3^) was similar to that of private vehicle BC (summer p50(IQR) = 1.8(1.6–5.9) µg/m^3^; winter p50(IQR) = 2.0(0.2–7.3) µg/m^3^). These comparisons emphasize the distinct air pollution profile of subways and the relative similarity of bus and private vehicle environments.

## Conclusion

In this paper we present concentrations for a wide variety of exhaust and non-exhaust traffic-related air pollutants for three Canadian bus transit systems. While the selection of bus routes did not employ statistical methods to ensure a representative sample, our sampling campaigns were extensive in both space and time. High ridership routes were selected which included the geographic extent of each bus network. As well, sampling was conducted during 15 summer and winter weekdays. Each day, three researchers concurrently sampled separate routes for three peak hours in the morning and evening. This equated to 540 h of sampling for each bus transit system. This dataset showed that bus commuting can contribute a high proportion of daily exposure to fine-fraction Fe and Ba as well as BC. We also found riding exposures to be lower for hybrid diesel/electric buses relative to buses of 1983–2003 make years. Owing to the magnitude of these differences and the frequency of which the 1983–2003 class was encountered during sampling, the replacement of these buses with hybrid diesel/electric buses was estimated to result in 4–18% lower riding exposures across the eight combinations of city and pollutant where hybrid diesel/electric buses were found to have lower concentrations than the 1983–2003 class.

Our results also featured several indications that emphasized the capacity of buses to self-pollute. Frequent braking and accelerating at intersections and bus stops are typical of many bus routes and provide opportunities for a bus’ own emissions to infiltrate into its cabin. In an analysis controlling for factors related to traffic, in-bus concentrations of several pollutants were found to be associated with bus type. This evidence suggests that a bus’ own exhaust emissions have an independent effect on the exposures of its passengers. Non-exhaust emissions may also enter a bus’ cabin more readily than exhaust emissions. While exhaust pipes were almost exclusively positioned on the roof at the rear of the buses, brake wear particles were emitted from each wheel. The high levels of the Ba and Fe brake markers in buses, relative to ambient, may also be an indicator of the capacity of buses to self-pollute [34].

In cities with limited subway systems, such as Ottawa, buses constitute the bulk of public transit infrastructure. In Toronto and Vancouver, bus transit networks are designed to complement the cross-city capacity of their extensive subway systems by providing transit from subway stations to stops within walking distance of of a majority of the city’s homes. This can result in bus transit ridership figures exceeding that of a city’s subway system. This is the case in Canada where the 2017 average weekday bus ridership exceeded that of the subway in Toronto (1,406,800 vs 877,300) and Vancouver (789,400 vs 472,100) [43]. This emphasizes the status of buses as a staple of public transit. Ensuring clean air in their waiting and riding environments is key to their sustainability. Future research should focus on assessing the impact of newer emission control technologies. Several cities are working towards the goal of featuring soot-free bus fleets. Such investments have the potential to save money after a period of 5–9 years [44].

## Supplementary information

Supplementary Information
