# Supplementary material for: Personal exposures to traffic-related air pollution in three Canadian bus transit systems: the Urban Transportation Exposure Study
Source: J Expo Sci Environ Epidemiol. 2020 Jul 16;31(4):628–40. doi: 10.1038/s41370-020-0242-2 (PMC8263338; doi:10.1038/s41370-020-0242-2)
Supplement: Supplementary file 1 — Supplementary Information [file 41370_2020_242_MOESM1_ESM.docx]

**Supporting Information for**

**Personal exposures to traffic-related air pollution in three Canadian bus transit systems: The Urban Transportation Exposure Study**

Keith Van Ryswyk*^1,2^, Greg Evans^2^, Ryan Kulka^1^, Liu Sun^1^, Kelly Sabaliauskas^2^, Mathieu Rouleau^1^, Angelos Anastasopolous^1^, Lance Wallace^3^, Scott Weichenthal^1,4^

^1^ Air Health Science Division, Health Canada, Ottawa, Ontario K1A 0K9, Canada

^2^ University of Toronto, Department of Chemical Engineering and Applied Chemistry, Toronto, Canada

^3^ Consultant, Santa Rosa, California 95409, United States

^4^ McGill University, Department of Epidemiology, Biostatistics, and Occupational Health, Montreal, Canada, H3A 1A2

*Corresponding Author

Email: [Keith.VanRyswyk@canada.ca](mailto:Keith.VanRyswyk@canada.ca)

Address:269 Laurier Ave West, Ottawa, Ontario, Canada, K1A 0K9

Telephone: 613-952-4582, Fax: 613-954-7612

Number of pages- 36

Number of figures- 17

Number of tables- 12

Table of Contents

[1. Supplementary Methodology Section 3](#_Toc37856721)

[1.1. Continuous air pollution monitoring (PM_2.5_, UFP, & BC) 3](#_Toc37856722)

[1.2 Integrated NO_2_, PM_2.5_ & PM_10_ methods 12](#_Toc37856723)

[3. Supplementary Results Section 14](#_Toc37856724)

[3. References 36](#_Toc37856725)

# 1. Supplementary Methodology Section

## 1.1. Continuous air pollution monitoring (PM_2.5_, UFP, & BC)

**Instruments**

Personal sampling backpacks contained instruments monitoring PM_2.5_ (TSI DustTrak 8520), UFP (CPC, TSI model 3007), and BC (AethLabs MicroAeth-51). The DustTrak is an optical light scattering monitor with a flow rate of 1.7 L/min equipped with a PM_2.5_ impactor and calibrated to a standard particle density. DustTrak units were checked for zero drift before and after each three-hour sampling session. While the DustTrak typically over predicts PM_2.5_ mass concentration due to the higher particle density of this standard, no adjustment of our data was applied (details in section 1.3). The CPC model 3007 counts all particles >10 nm in aerodynamic diameter. Although this includes particles above the ultrafine particle upper limit (100 nm) these are normally so few in number that they do not impact the number count significantly; therefore we describe its output as UFP. If particles <10 nm are present, the output will be an underestimate. The microaetholometer-51 measures the absorbance of infrared light (880nm) of particles which accumulate on filter tickets.

**Calibration, Quality Assurance, and Quality Control**

Factory calibration of the continuous monitoring units for PM_2.5_, UFP, and BC was conducted before each seasonal sampling campaign. After each seasonal sampling campaign, units were co-located for an inter-comparative sampling session. This involved the concurrent monitoring by all units for at least one hour of a small burning event. These data were used to calculate instrument precision, bias, and overall limit of detection as per Wallace et al (2011)^1^. Table S1 provides the descriptive statistics of these co-location sessions along with the limit of detection, bias and bias-corrected precision estimates.

**Comparison between nephelometric and gravimetric PM_2.5_ methods**

This study included one continuous (TSI DustTrak) and one gravimetric (PEMs: see section 2 below) method for quantifying PM_2.5_ exposure. The DustTrak was chosen for its high temporal resolution and the gravimetric method provided particles for elemental analyses. Their colocation during the study also allowed for their comparison as a measure of quality assurance. There were 9 gravimetric PM_2.5_ samples deployed in each combination of city and season (n=6). DustTrak data representing at least 75% of the 30-hour gravimetric sampling period was required to be included in this analysis. Due to invalid PM samples and insufficient DustTrak data, 45 of the possible 54 data points were available for this analysis. A poor correlation between these methods was found when examining the data as a whole. This prompted an examination within each city-season group. Results are presented in Figure S1A-F. Typically, the DustTrak 8520 over predicts gravimetric PM_2.5_ methods by a factor of 2-3. This was the case for the summer seasons of all cities and the winter season in Toronto. Across the six groups of city and season, R^2^ values ranged from 0-79%. Within each city, some PM samples may be representing routes where there is a higher exposure to non-tailpipe traffic emissions in below-grade/indoor bus stations. Fieldwork error was also considered as a source of the lack of consistency between these methods. However, it is important to note that the tools, methods, protocols and technicians used in this fieldwork were the same used in the subway component of the Urban Transportation Exposure study (UTES) which was conducted a week before and found excellent agreement between these two methods (R2 = 0.89; slope = 0.96)^2^. The heterogeneity in these continuous: gravimetric relationships suggest variation in the density of PM_2.5_ in the bus environments of these cities and perhaps season-to-season. The small sample size within these six groups also hinders the ability to confidently quantify the relationship between continuous and gravimetric and derive a scaling factor for the DustTrak data. Therefore, the DustTrak values presented are unadjusted and we use them solely for relative comparisons between bus and bus stop types within each city.

| **Table S1: Descriptive statistics of co–location sampling conducted after each sampling session with limits of detection, bias and precision.** | | | | | | | | | | | | | | |  |
| --- | --- | --- | --- | --- | --- | --- | --- | --- | --- | --- | --- | --- | --- | --- | --- |
|  | Instrument  (pollutant & units) | Session* | N units | time | mean |  | limit of detection |  | Bias** | |  | Bias–Corrected Precision (%) | |  | |
|  |  |  |  | (hours) |  |  |  |  | median | range |  | Median | Range |  | |
|  | DustTrak  (PM_2.5_) (µg/m^3^) | T&O summer | 7 | 9 | 2.79 |  | 3.2 |  | 1 | 0.6 - 1.4 |  | 20.8 | 14 - 31 |  | |
|  |  | T&O winter | 6 | 22.7 | 38.5 |  | 22 |  | 1.1 | 1.0 - 1.2 |  | 27.1 | 16 - 38 |  | |
|  |  | V winter | 6 | 1.2 | 10.3 |  | 3 |  | 1.3 | 1.1 – 2.2 |  | 38.7 | 25 – 75 |  | |
|  |  | V summer | 7 | 1.5 | 12.3 |  | 3.3 |  | 1.1 | 0.7 – 1.4 |  | 21.1 | 18 - 34 |  | |
|  | CPC  (UFP) (pts/cm^3^) | T&O summer | 7 | 8.9 | 4527 |  | 1573 |  | 1.1 | 0.5 – 1.6 |  | 19 | 8 – 28 |  | |
|  |  | T&O winter | 6 | 3.4 | 11096 |  | 1211 |  | 1 | 0.6 – 1.3 |  | 21.5 | 10 – 44 |  | |
|  |  | V winter | 7 | 0.8 | 6963 |  | 3581 |  | 1.1 | 1.0 – 1.1 |  | 10.9 | 7 – 17 |  | |
|  |  | V summer | 8 | 1.5 | 6749 |  | 2078 |  | 1 | 0.9 – 1.2 |  | 8.2 | 5 - 40 |  | |
|  | MicroAeth 51  (Black Carbon) (ng/m^3^) | T&O summer | 8 | 12.9 | 1579 |  | 208 |  | 1.1 | 0.9 – 1.1 |  | 6.6 | 4 – 33 |  | |
|  |  | T&O winter | 4 | 3.1 | 1584 |  | 100 |  | 1.1 | 0.8 – 1.4 |  | 4.6 | 2 – 24 |  | |
|  |  | V winter | 5 | 1.6 | 151 |  | 158 |  | 0.8 | -2.0 – 3.6 |  | 28.2 | 18 – 37 |  | |
|  |  | V summer | 5 | 1.8 | 221 |  | 302 |  | 1.5 | -2.0 – 2.1 |  | 56 | 31 - 83 |  | |
| *T&O = Toronto & Ottawa, V = Vancouver; **Referent is the mean of all instruments. | | | | | | | | | | | |  |  |  | |

**
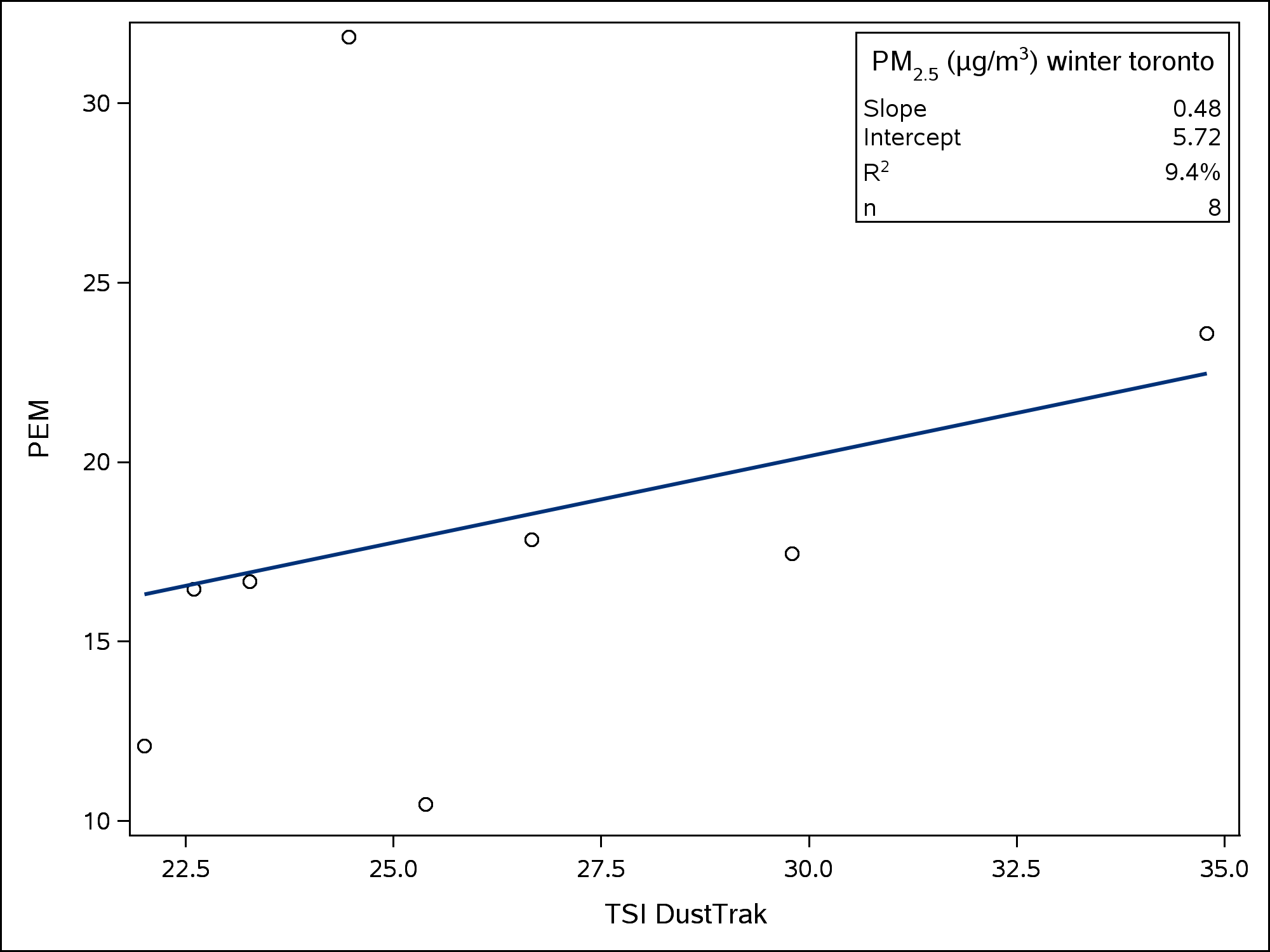
**

**Figure S1A: comparison of PEM to TSI Dusttrak 8520 in Toronto winter campaign.**

**
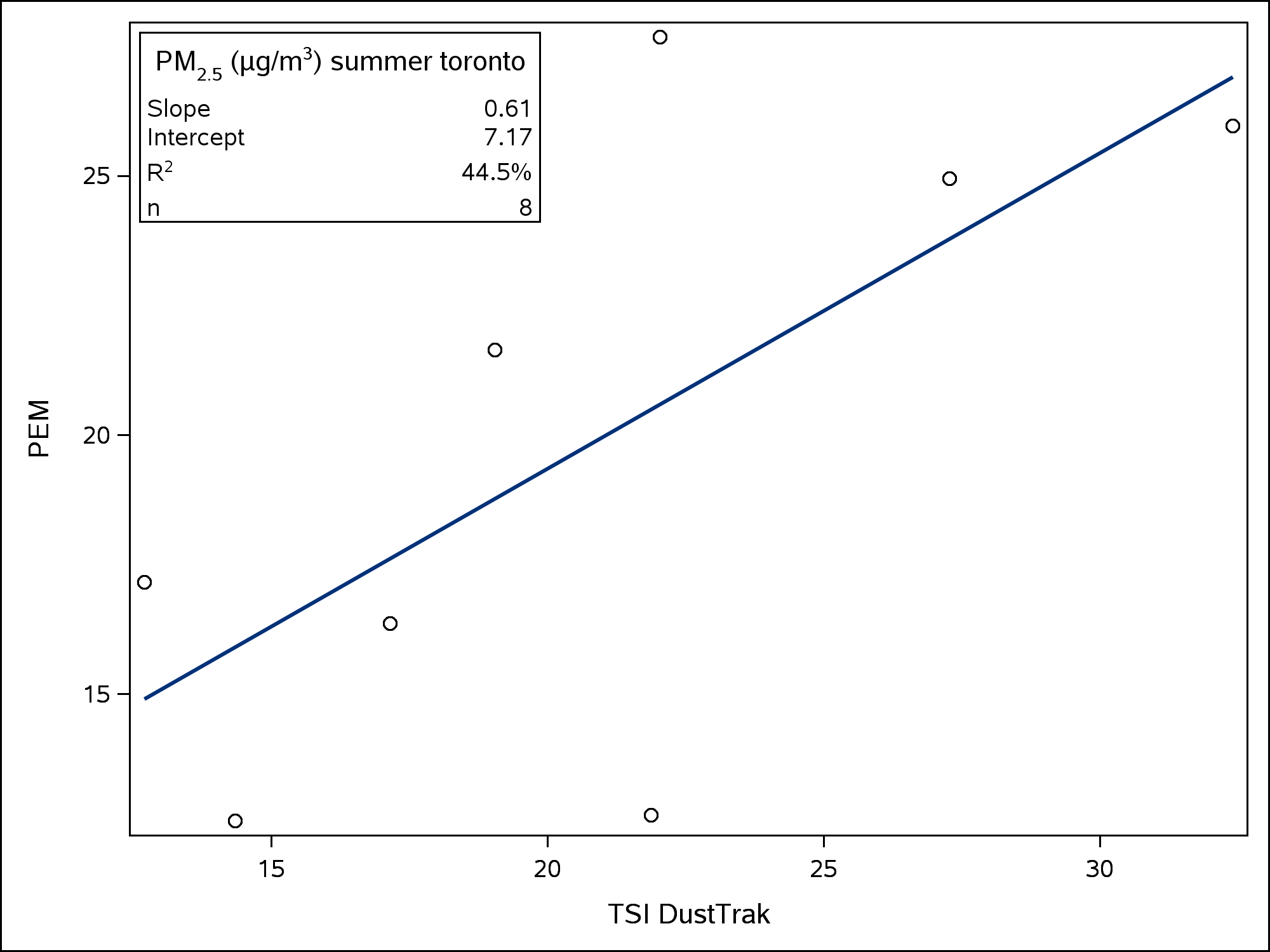
**

**Figure S1B: comparison of PEM to TSI Dusttrak 8520 in Toronto summer campaign.**

**
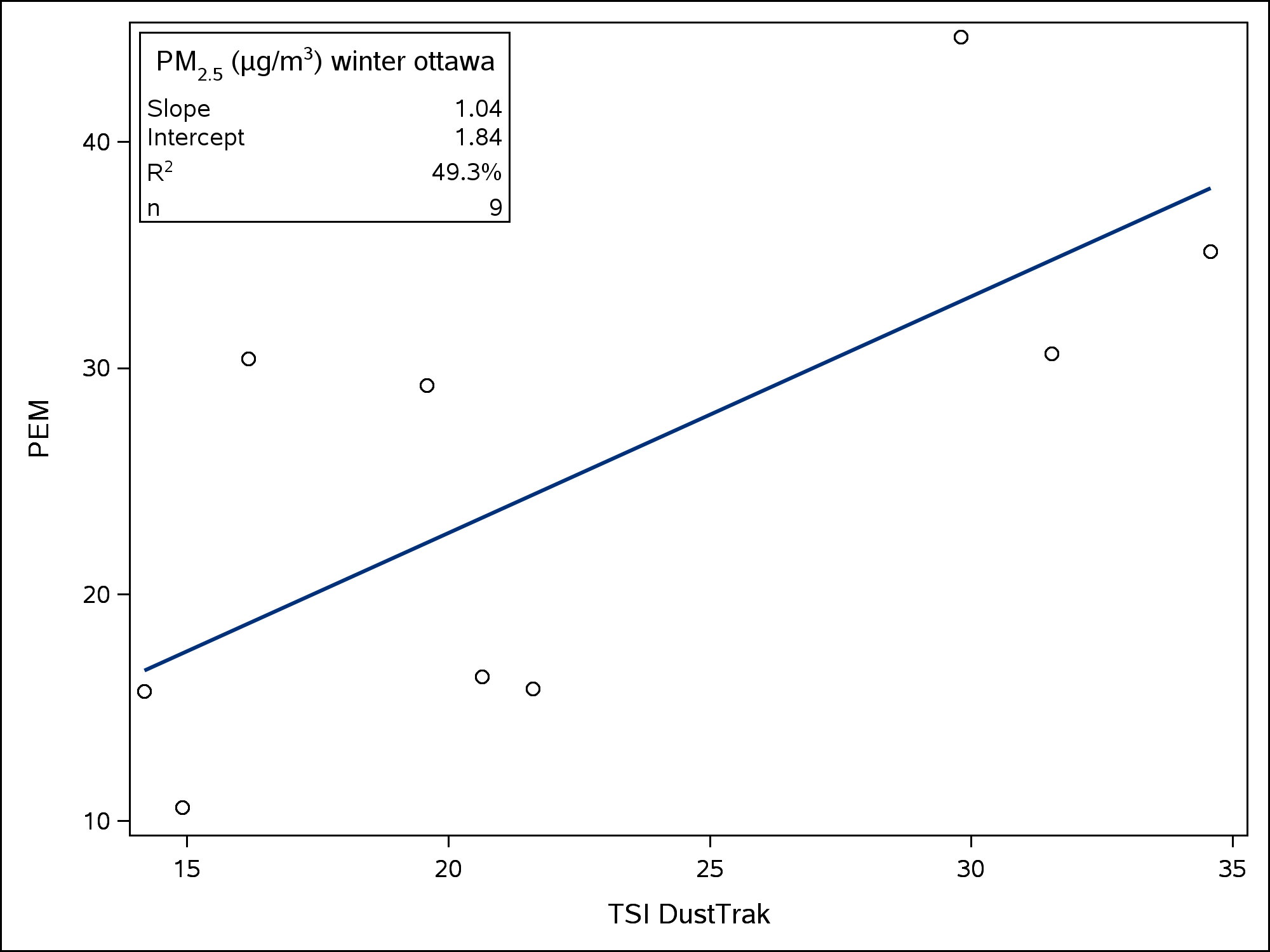
**

**Figure S1C: Comparison of PEM to TSI Dusttrak 8520 in Ottawa winter campaign.**

**
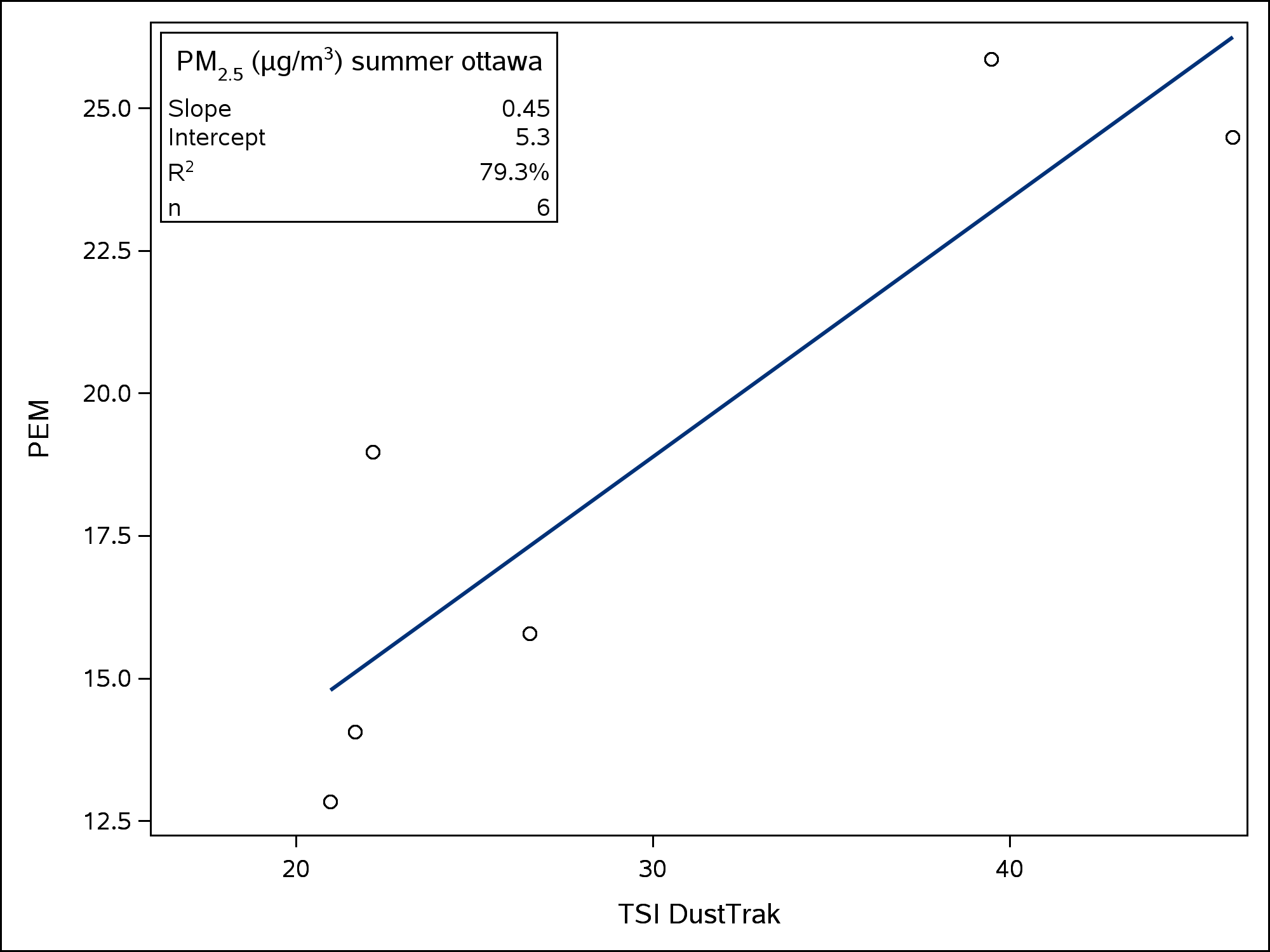
**

**Figure S1D: Comparison of PEM to TSI Dusttrak 8520 in Ottawa summer campaign.**

**
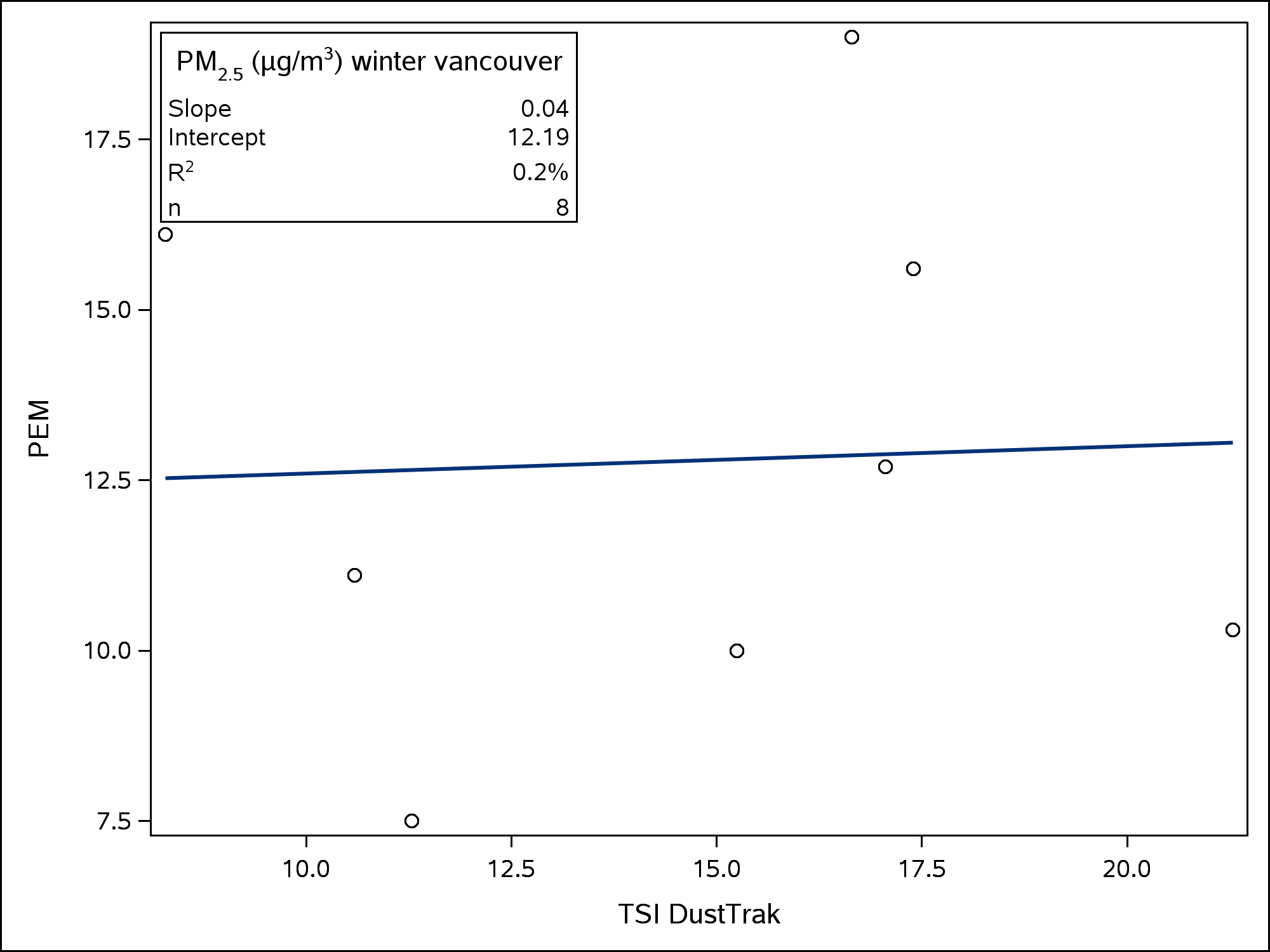
**

**Figure S1E: Comparison of PEM to TSI Dusttrak 8520 in Vancouver winter campaign.**

**
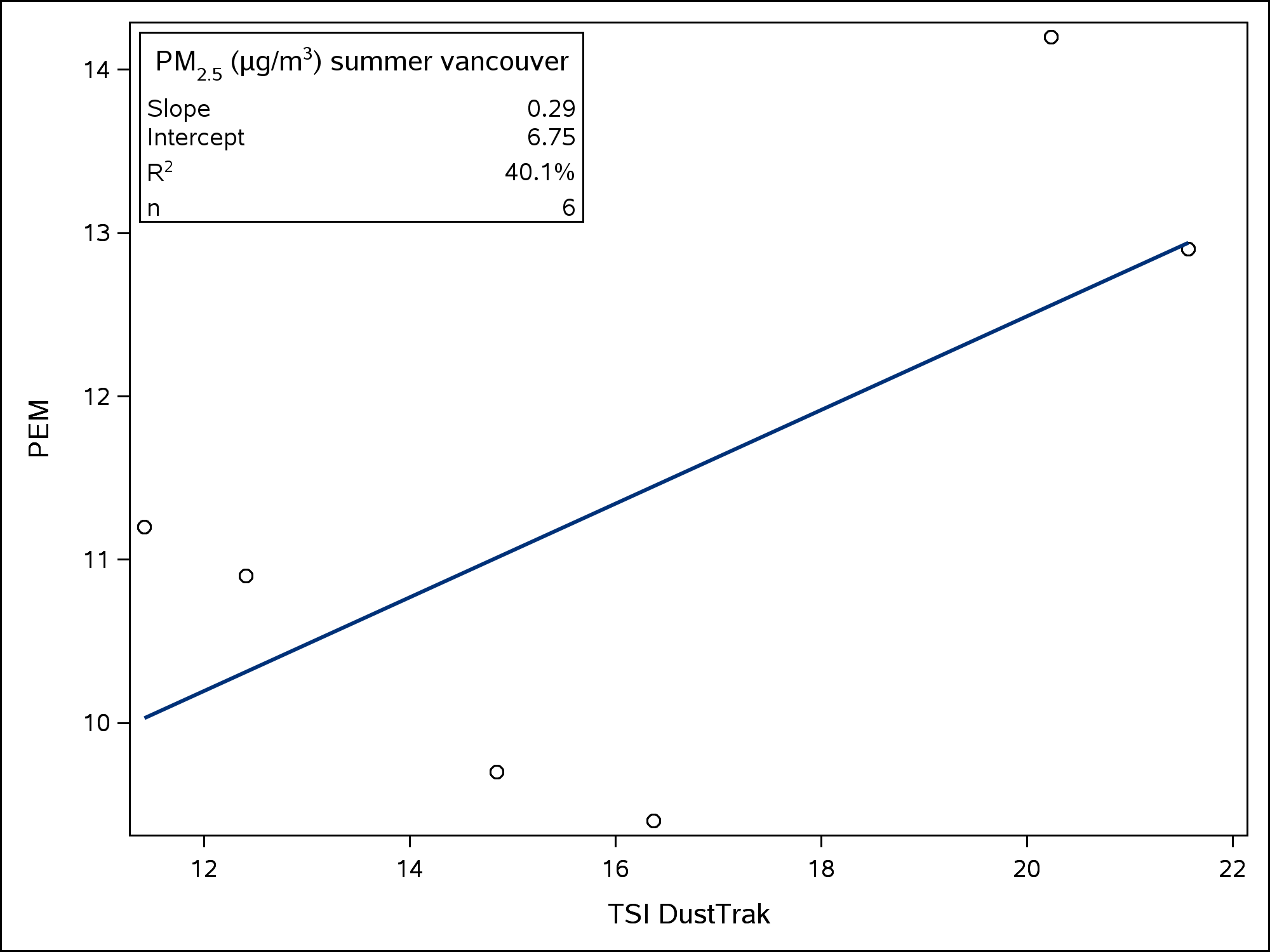
**

**Figure S1F: Comparison of PEM to TSI Dusttrak 8520 in Vancouver summer campaign.**

## 1.2 Integrated NO_2_, PM_2.5_ & PM_10_ methods

Personal NO_2_ exposures were measured using passive samplers (Ogawa & Company, USA) in Ottawa and Toronto in the winter and both seasons in Vancouver. These passive badges were worn in the breathing zone of the technicians and were analysed according to Ogawa standard protocols. Each sample was integrated over each three-hour sampling session. Gravimetric PM_2.5_ and PM_10_ samples were collected using Personal Environmental Monitors (PEMs) of the Chempass System (Chempass System R&P/Thermo) which feature particle size selective inlets and impaction plates. Particles were collected on 37mm Teflon filters. Sample flow (4 lpm) was provided by a personal sampling pump (SKC Leland). Integrated PM samples reflected the ten 3-hour sampling periods conducted by a technician over a week. This provided 18 PM_2.5_ and 18 PM_10_ gravimetric samples for each city. Sample flows were measured at the beginning and end of each 3-hour sampling session. Between sessions, samples were stored in sealed bags. Samples operating within 25% of the targeted 30 hours of sampling were deemed valid. As well, samples required an average flow rate within 10% of the target 4 litres per minute (lpm). The integration of gravimetric samples over one week of sampling (30 hours) was done to ensure detectable levels of the elemental components of the PM samples. Gravimetric analyses were done in accordance with EPA guidelines. Inductively Coupled Plasma-Mass Spectrometry (ICP-MS) quantified 36 elemental concentrations of PM samples. Field blanks for all integrated samples were carried in back packs and analysed along with samples. Median field blank concentrations found to be greater than the lab detection limit were used to blank-correct.


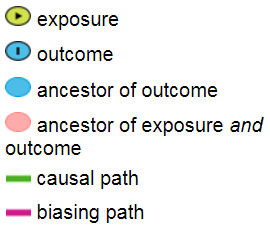

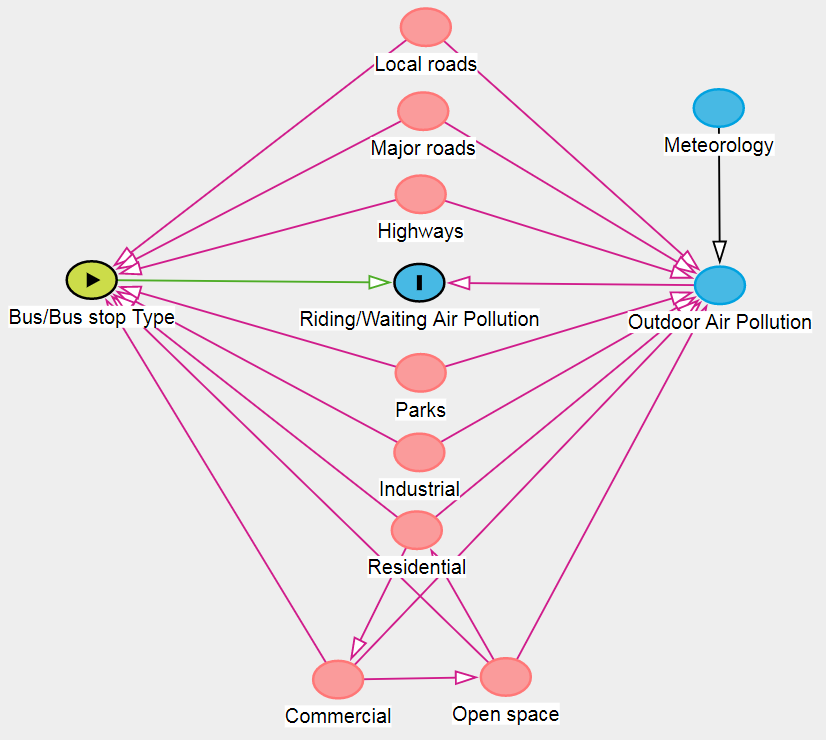


**Figure S2. Directed acyclic graph for estimating the direct effect of bus and bus stop type on riding and waiting exposures, respectively.**

# 3. Supplementary Results Section

The spatial variability of the continuously monitored pollutants for Toronto, Ottawa, and Vancouver can be seen in Figures S3-5 (PM_2.5_), Figures S6-8 (UFP) and Figures S9-11 (BC). Points represent mean exposures for waiting periods at bus stops and stations. Riding data were averaged over each 50m of bus route segments. Descriptive statistics of PM_2.5_, UFP, BC, and NO_2_ exposures by city are presented in Table S3 along with tests for seasonality (α<0.05). While PM_2.5_ seasonality was noted in each city’s bus exposures, higher levels were found in winter for Toronto, while the opposite trend was seen in Ottawa and Vancouver. Winter UFP levels were significantly higher in summer in the bus environments of all three cities. However, the magnitude of this difference was highest for Vancouver followed by Ottawa then Toronto. Black carbon levels were higher in summer for Ottawa and Toronto while no seasonality in bus BC levels was seen in Vancouver. Winter NO_2_ was comparable in the three cities. Summer NO_2_ data was significantly lower than winter. Summer NO_2_ was unavailable for Ottawa and Toronto. Levels of waiting versus riding for continuously monitored pollutants are presented in Table S4. In all three cities, waiting UFPs exposures were found to be significantly higher then riding exposures while PM_2.5_ and BC were similar across waiting and riding status. The mass concentration and elemental content of the gravimetric PM_2.5_ and PM_10_ samples collected in the bus transit systems of Toronto, Ottawa, and Vancouver are presented in Table S7A, S7B, and S7C, respectively. The ICP-MS analyses accounted for 21.3%, 20.2%, and 14.9% of the PM_2.5_ mass and 36.7%, 32.8%, and 26.5% for PM_10_ mass in Toronto, Ottawa, and Vancouver, respectively (Figure S12). In all three cities, PM was enriched with elements related to road and/or sea salt (Na and Cl), road dust (Fe, Ca, and Si), tire wear (Zn), and primary vehicle and brake emissions (Ba and Cu). Vancouver PM demonstrated a distinct elemental profile in that it had higher enrichments of Si and Al while having less Cl. Si is the traditional marker for crustal component of PM (earth/soil). Therefore, this may be reflecting a regional difference in crustal composition, where Si is higher on west coast than in central region.

| **Table S2. Number and duration of riding and waiting sampling sessions.** | | | | | | | | |  |
| --- | --- | --- | --- | --- | --- | --- | --- | --- | --- |
|  | City | status | duration (mins) | | | | | |  |
|  |  |  | n | mean | stddev | min | median | max |  |
|  | Toronto | riding | 995 | 17.3 | 12.8 | 1 | 15 | 111 |  |
|  |  | waiting | 958 | 7.6 | 7.7 | 1 | 6 | 149 |  |
|  | Ottawa | riding | 1699 | 8.0 | 7.8 | 1 | 5 | 78 |  |
|  |  | waiting | 1774 | 8.6 | 6.6 | 1 | 7 | 60 |  |
|  | Vancouver | riding | 1408 | 14.1 | 9.6 | 1 | 11 | 56 |  |
|  |  | waiting | 1467 | 8.1 | 6.3 | 1 | 7 | 105 |  |
|  |  |  |  |  |  |  |  |  |  |


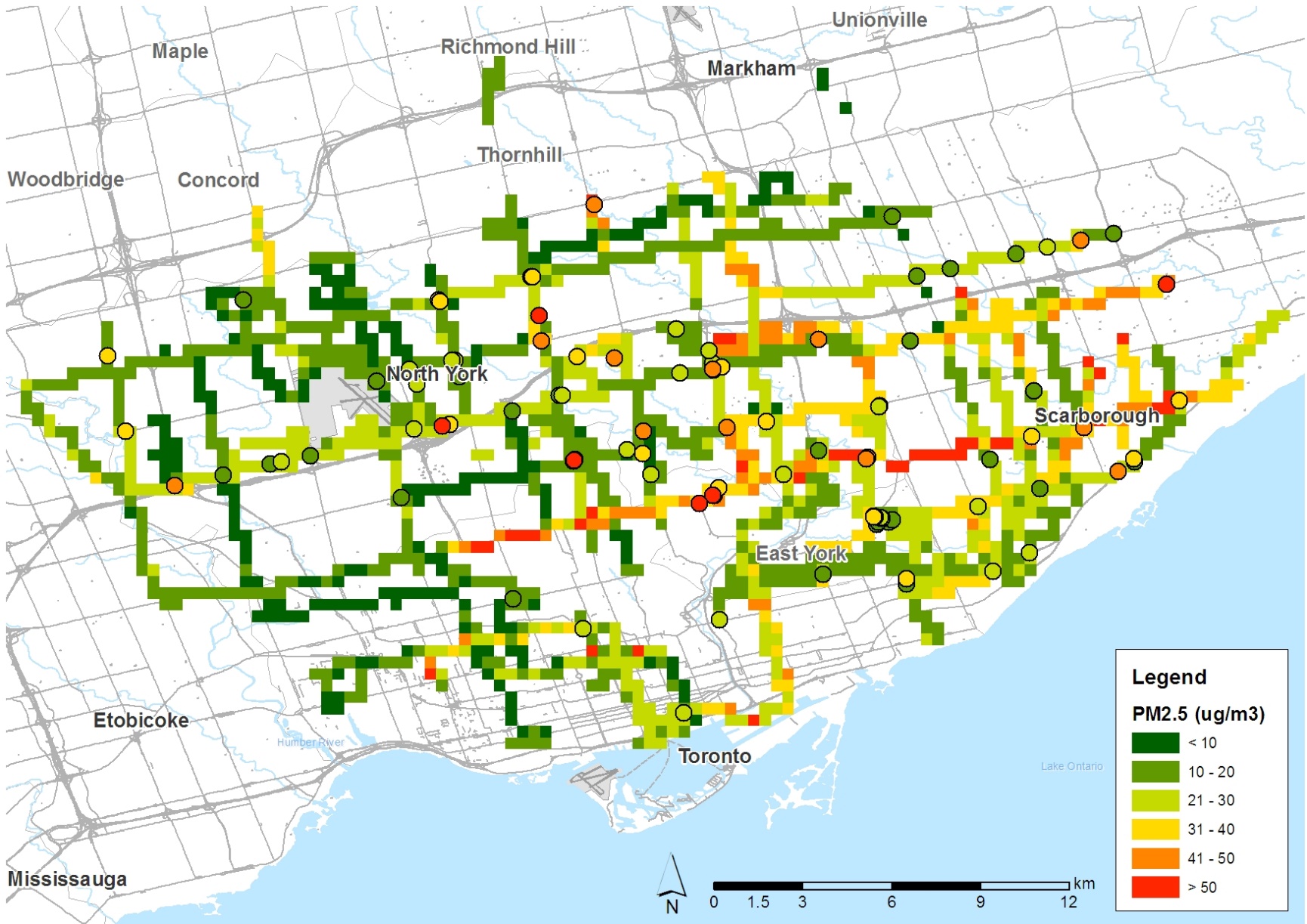


**Figure S3: Mean PM_2.5_ levels during riding (squares) and waiting (circles) in Toronto, Canada**


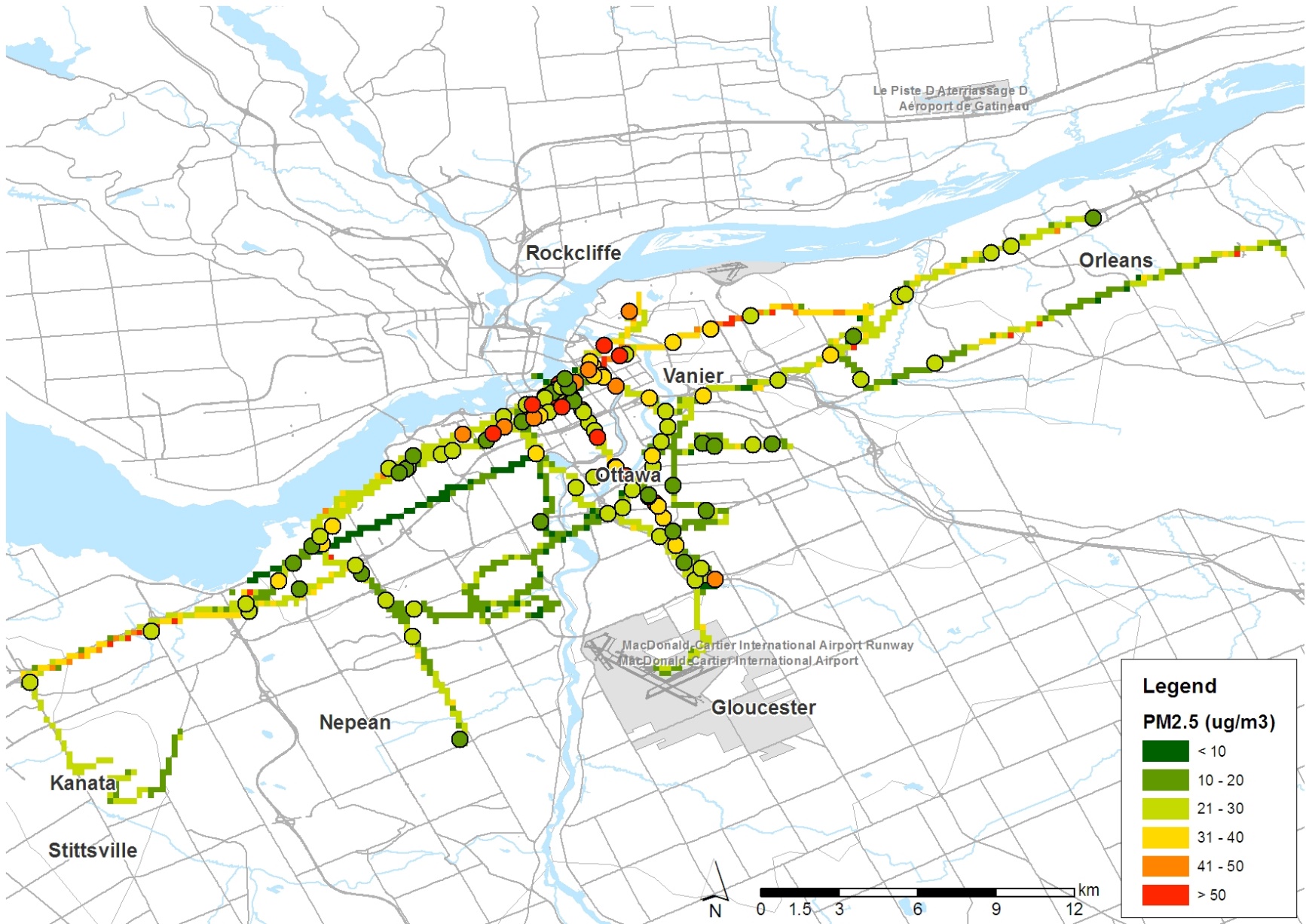


**Figure S4: Mean PM_2.5_ levels during riding (squares) and waiting (circles) in Ottawa, Canada.**


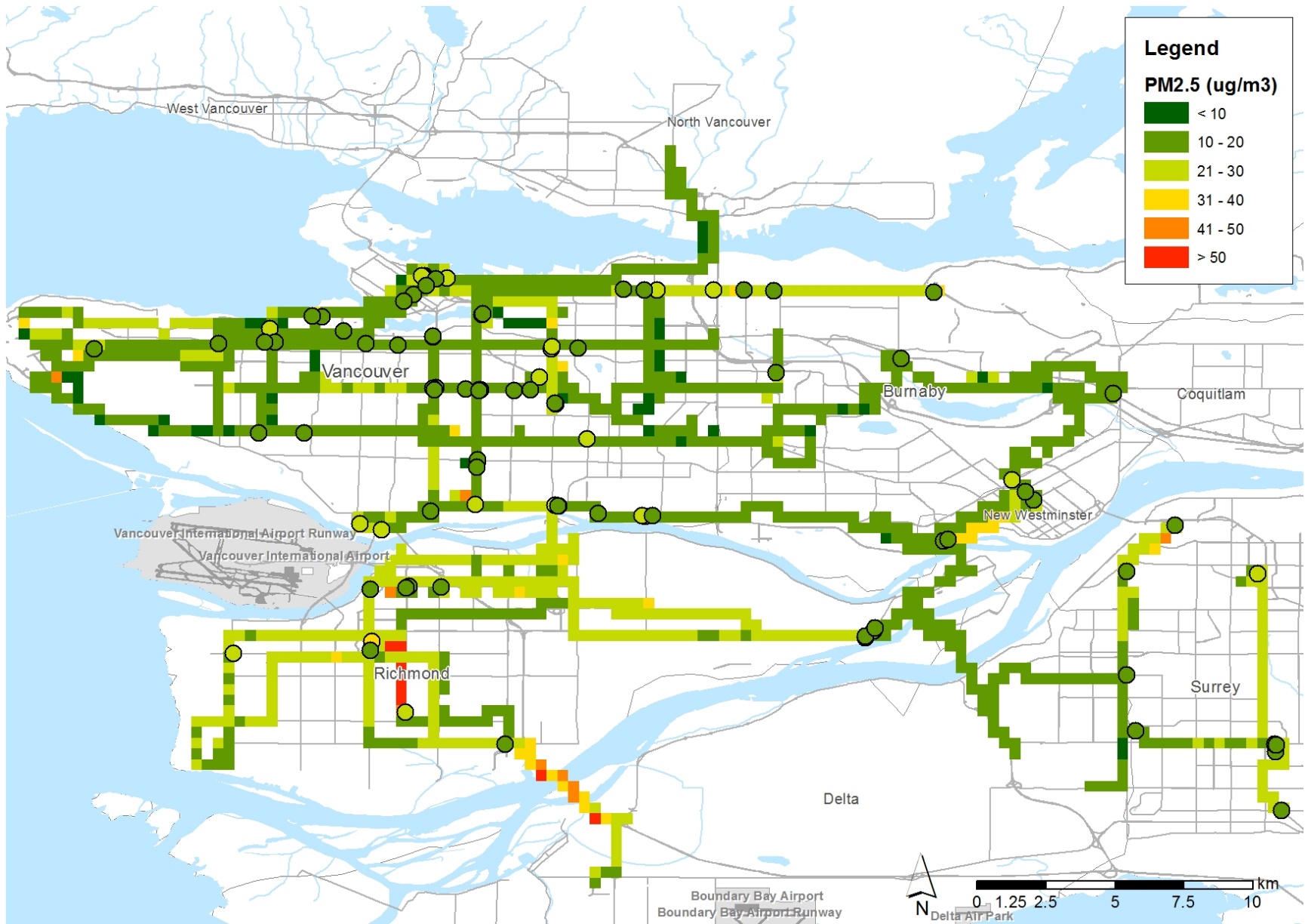


**Figure S5: Mean PM_2.5_ levels during riding (squares) and waiting (circles) in Vancouver, Canada.**


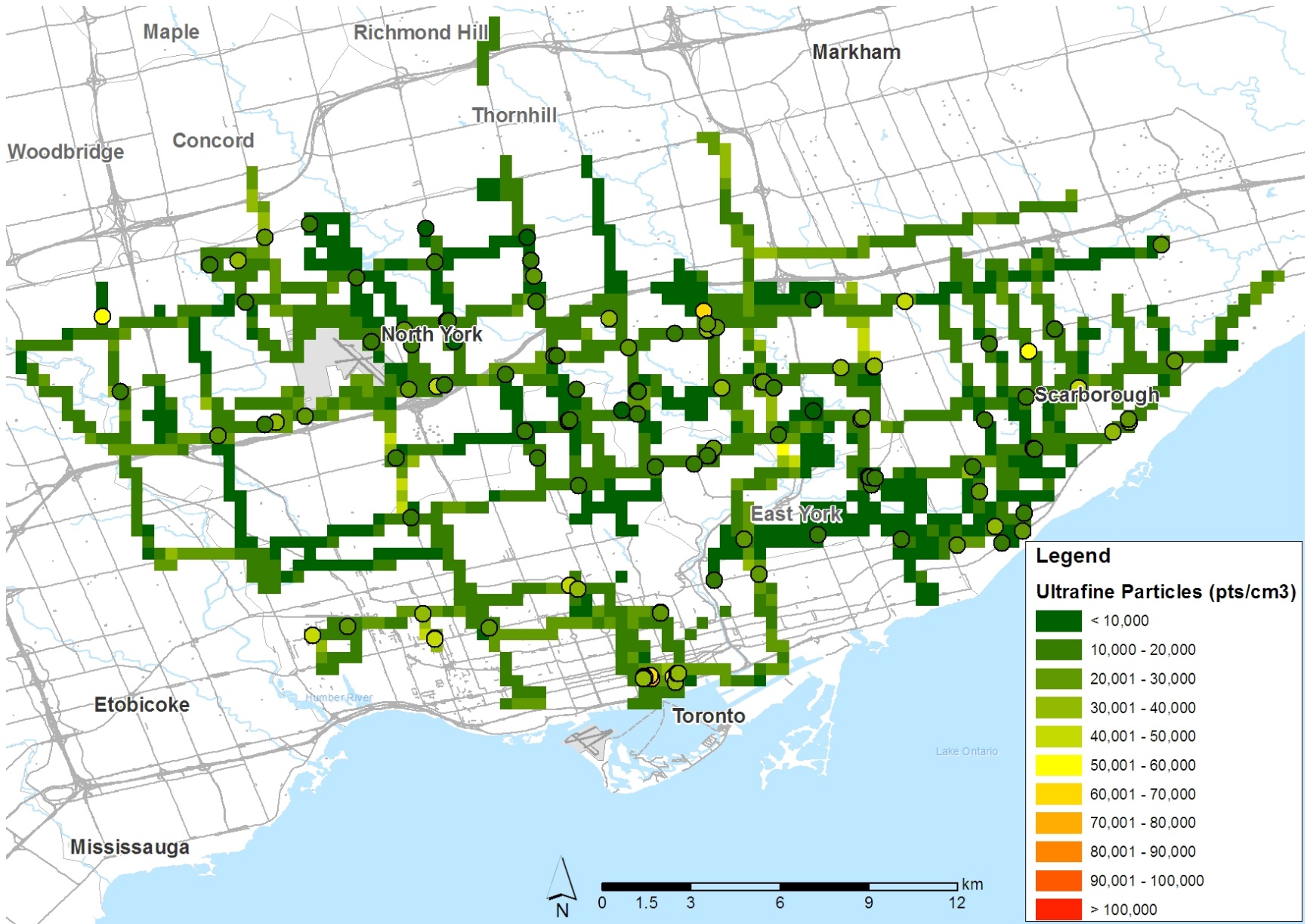


**Figure S6: Mean UFP levels during riding (squares) and waiting (circles) in Toronto, Canada.**


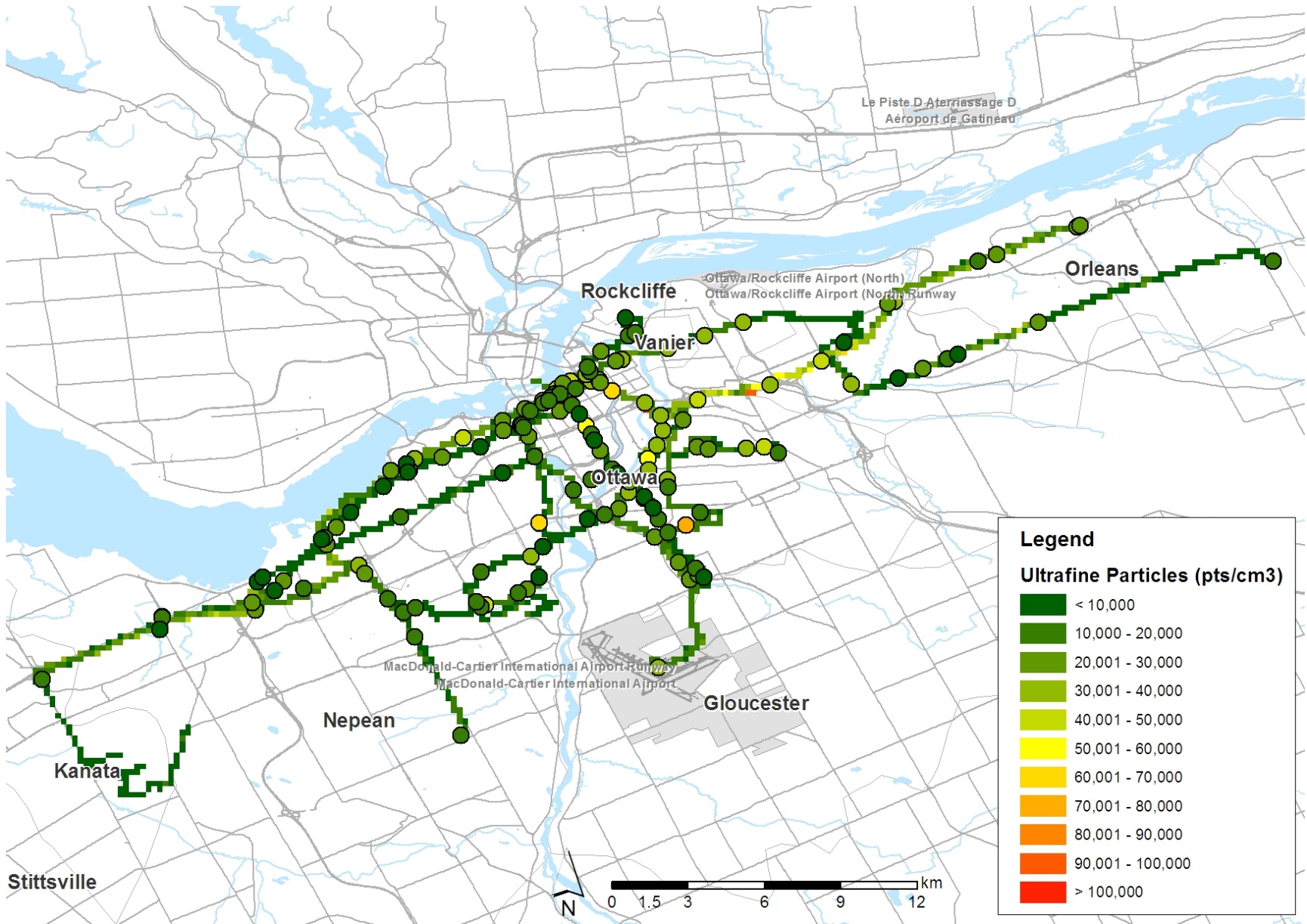


**Figure S7: Mean UFP levels during riding (squares) and waiting (circles) in Ottawa, Canada**


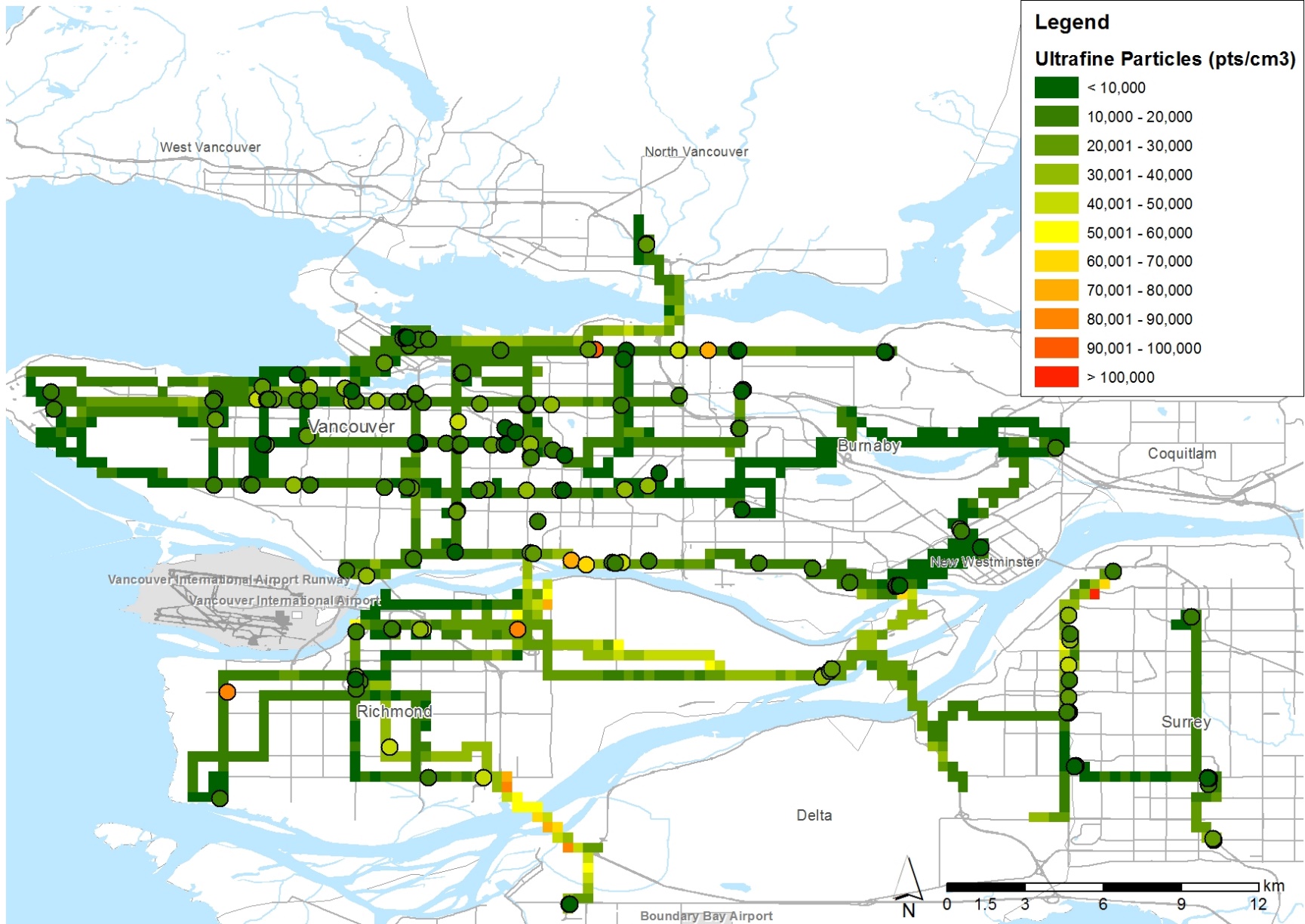


**Figure S8: Mean UFP levels during riding (squares) and waiting (circles) in Vancouver, Canada.**


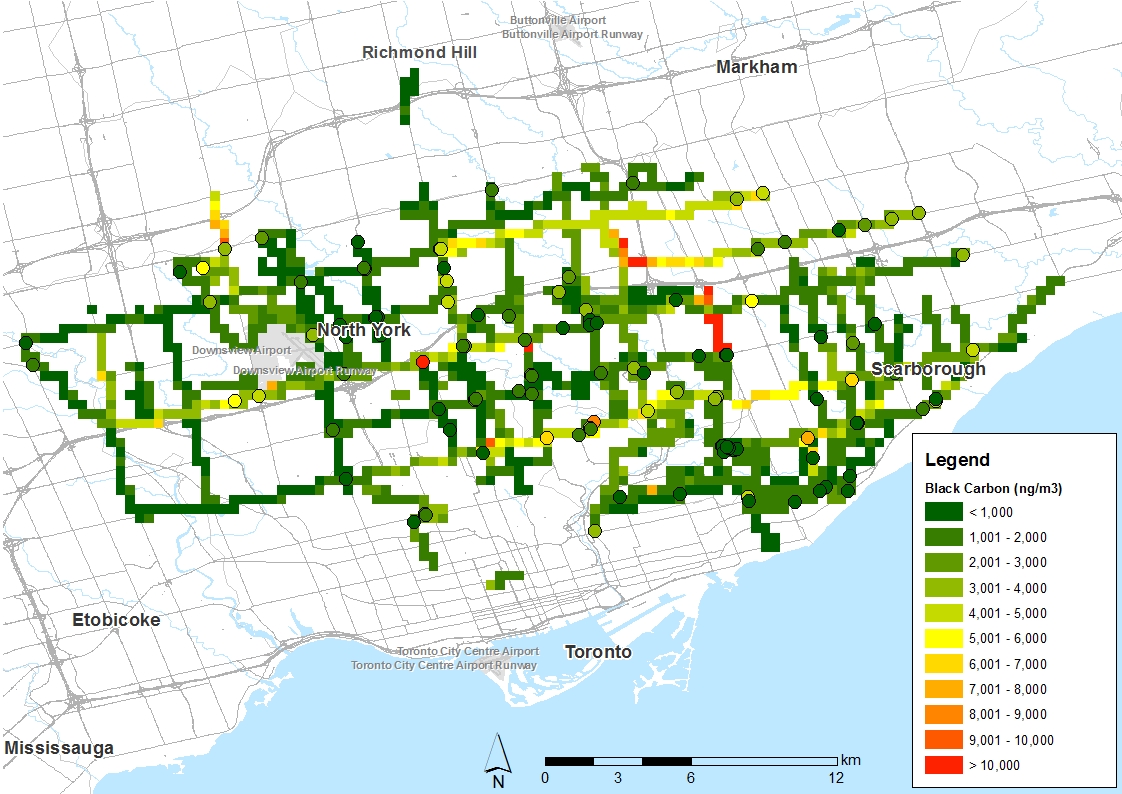


**Figure S9: Mean BC levels during riding (squares) and waiting (circles) in Toronto, Canada.**


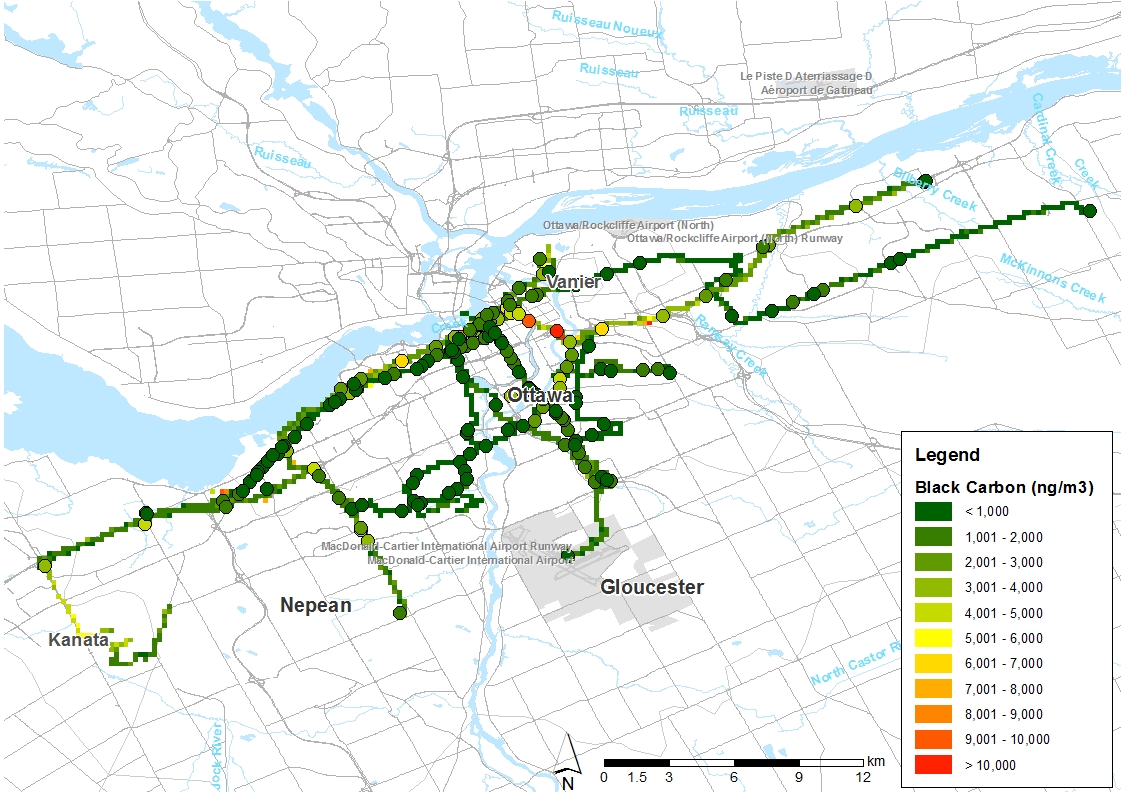


**Figure S10: Mean BC levels during riding (squares) and waiting (circles) in Ottawa, Canada.**


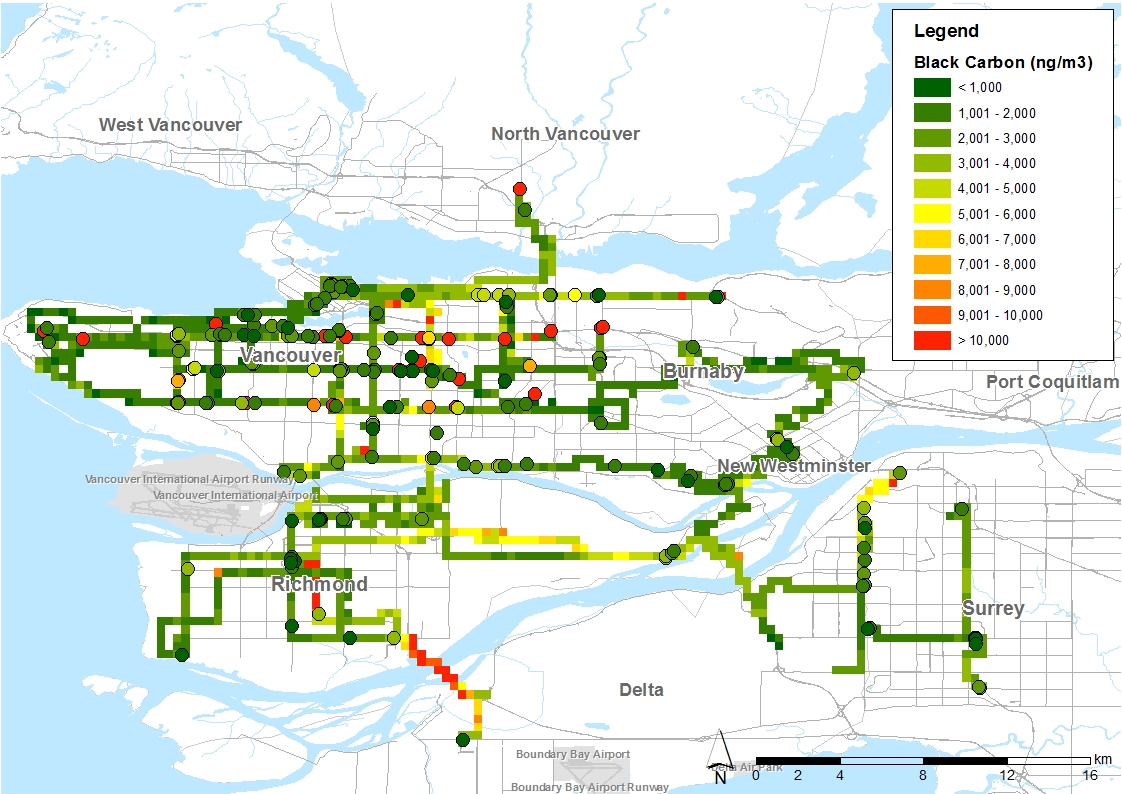


**Figure S11: Mean BC levels during riding (squares) and waiting (circles) in Vancouver, Canada.**

| **Table S3. Descriptive statistics by city and season for monitored traffic-related air pollutants.** | | | | | | | |
| --- | --- | --- | --- | --- | --- | --- | --- |
|  |  |  |  |  |  |  |  |
| Pollutant | city | season | n | mean | SD | median | IQR |
| PM_2.5_^†^  (µg/m^3^) | Toronto | summer** | 627 | 23.2 | 17.6 | 18.8 | 12.2 - 27.5 |
|  |  | winter | 1244 | 26.9 | 18.6 | 21.2 | 12.7 - 37.9 |
|  | Ottawa | summer** | 1624 | 27.3 | 21.7 | 22.0 | 12.5 - 35.2 |
|  |  | winter | 1747 | 22.3 | 19.3 | 16.0 | 9.3 - 28.7 |
|  | Vancouver | summer** | 1380 | 19.1 | 10.9 | 17.1 | 12.0 - 25.0 |
|  |  | winter | 1476 | 13.9 | 9.6 | 11.5 | 7.6 - 17.6 |
| Ultrafine Particles  (10^3^ pts/cm^3^) | Toronto | summer** | 361 | 17.6 | 11.6 | 15.0 | 8.6 - 24.2 |
|  |  | winter | 1105 | 21.5 | 15.6 | 17.3 | 10.7 - 27.7 |
|  | Ottawa | summer** | 1463 | 13.9 | 10.9 | 10.9 | 6.4 - 17.7 |
|  |  | winter | 1716 | 32.2 | 33.0 | 22.3 | 11.2 - 41.6 |
|  | Vancouver | summer** | 1342 | 6.0 | 4.7 | 4.8 | 3.0 - 7.5 |
|  |  | winter | 1407 | 29.4 | 23.2 | 23.4 | 13.8 - 38.6 |
| Black Carbon  (µg/m^3^) | Toronto | summer** | 617 | 5.6 | 8.2 | 3.2 | 1.7 - 5.9 |
|  |  | winter | 966 | 1.7 | 2.1 | 1.1 | 0.6 - 2.1 |
|  | Ottawa | summer** | 1592 | 4.1 | 4.2 | 2.8 | 1.4 - 5.3 |
|  |  | winter | 1710 | 1.5 | 1.9 | 0.9 | 0.5 - 1.8 |
|  | Vancouver | summer | 1106 | 2.3 | 2.3 | 1.7 | 1.0 - 2.9 |
|  |  | winter | 1384 | 2.4 | 2.1 | 1.8 | 1.1 - 3.1 |
| NO_2_^±^  (ppb) | Toronto | summer | 0 | - | - | - | - |
|  |  | winter | 74 | 59 | 53 | 51 | 17-75 |
|  | Ottawa | summer | 0 | - | - | - | - |
|  |  | winter | 83 | 48 | 78 | 8 | 7-59 |
|  | Vancouver | summer** | 83 | 37 | 61 | 25 | 18-34 |
|  |  | winter | 81 | 50 | 44 | 39 | 17-66 |
| *p<0.05; **p<0.0001; ^†^unscaled DustTrak data; ^±^NO_2_ samples integrated over 3-hour sampling periods.  F | | | | | | | |

| **Table S4. Descriptive statistics by city and waiting/riding for particulate pollutants monitored in bus transit systems.** | | | | | | | | | |
| --- | --- | --- | --- | --- | --- | --- | --- | --- | --- |
| pollutant | city | mode | n |  | mean | SD |  | median | IQR |
| PM_2.5_^†^  (µg/m^3^) | Toronto | riding** | 952 |  | 23.4 | 16.0 |  | 19.0 | 12.6 - 30.1 |
|  |  | waiting | 919 |  | 27.9 | 20.3 |  | 22.4 | 12.5 - 39.2 |
|  | Ottawa | riding | 1650 |  | 24.9 | 21.1 |  | 19.0 | 11.1 - 32.1 |
|  |  | waiting | 1721 |  | 24.5 | 20.2 |  | 18.4 | 9.8 - 32.4 |
|  | Vancouver | riding** | 1399 |  | 18.0 | 11.5 |  | 15.5 | 10.0 - 23.2 |
|  |  | waiting | 1457 |  | 15.0 | 9.3 |  | 13.1 | 8.5 - 19.1 |
| Ultrafine particles (10^3^ pts/cm^3^) | Toronto | riding** | 739 |  | 15.9 | 11.4 |  | 13.2 | 8.3 - 20.2 |
|  |  | waiting | 727 |  | 25.3 | 16.3 |  | 21.8 | 14.1 - 31.4 |
|  | Ottawa | riding** | 1556 |  | 19.4 | 20.4 |  | 12.9 | 7.2 - 24.3 |
|  |  | waiting | 1623 |  | 27.9 | 31.5 |  | 17.4 | 9.5 - 34.3 |
|  | Vancouver | riding* | 1349 |  | 16.8 | 19.3 |  | 10.2 | 4.7 - 22.3 |
|  |  | waiting | 1400 |  | 19.1 | 21.7 |  | 10.4 | 4.6 - 26.6 |
| BC (µg/m^3^) | Toronto | riding* | 820 |  | 2.9 | 3.4 |  | 1.8 | 0.9 - 3.4 |
|  |  | waiting | 763 |  | 3.6 | 7.4 |  | 1.6 | 0.7 - 3.5 |
|  | Ottawa | riding | 1618 |  | 2.7 | 2.9 |  | 1.7 | 0.8 - 3.4 |
|  |  | waiting | 1684 |  | 2.8 | 3.9 |  | 1.5 | 0.7 - 3.2 |
|  | Vancouver | riding** | 1241 |  | 2.7 | 2.2 |  | 2.1 | 1.4 - 3.3 |
|  |  | waiting | 1249 |  | 2.0 | 2.2 |  | 1.4 | 0.8 - 2.5 |
| Test for a significant difference between waiting and riding exposures; *p<0.05; **p<0.0001;^†^unscaled DustTrak data | | | | | | | | | |

| **Table S5. Riding exposures of PM2.5, UFP, and BC by city and bus type.** | | | | | | |
| --- | --- | --- | --- | --- | --- | --- |
| Pollutant | City | bus type | n | mean(SD) | median(p25-p75) | |
| PM_2.5_^†^ | Toronto | 1983-2003 diesel | 147 | 24.4 (15.2) | 20.8 (30.1-86.8) | |
| (µg/m^3^) |  | 2004-2006 diesel | 269 | 28.8 (17.1) | 24.3 (37.7-107.8) | |
|  |  | 2007- diesel | 107 | 16.3 (9.9) | 13.1 (20.4-52.2) | |
|  |  | hybrid diesel/electric | 407 | 21.3 (15.7) | 17.4 (26.9-177.9) | |
|  | Ottawa | 1983-2003 diesel | 415 | 27.2 (22.4) | 21.3 (35.4-172.6) | |
|  |  | 2004-2006 diesel | 276 | 27.9 (21.6) | 22.7 (34.8-169.7) | |
|  |  | 2007- diesel | 598 | 24.1 (22.0) | 18.3 (29.8-275.2) | |
|  |  | hybrid diesel/electric | 242 | 19.3 (15.6) | 15.4 (25.6-121.7) | |
|  | Vancouver | 1983-2003 diesel | 405 | 23.6 (11.7) | 21.5 (29.0-87.2) | |
|  |  | 2004-2006 diesel | 57 | 19.4 (11.2) | 17.2 (22.7-70.8) | |
|  |  | 2007- diesel | 366 | 16.0 (11.6) | 13.3 (19.5-123.3) | |
|  |  | hybrid diesel/electric | 219 | 14.4 (9.5) | 12.4 (17.9-59.2) | |
|  |  | electric* | 313 | 14.5 (9.0) | 12.8 (18.2-95.4) | |
| Ultrafine | Toronto | 1983-2003 diesel | 79 | 20.6 (12.6) | 18.4 (26.0-73.1) | |
| Particles (103/cm3) |  | 2004-2006 diesel | 243 | 14.4 (12.9) | 10.6 (18.0-110.7) | |
| (10^3^/cm^3^) |  | 2007- diesel | 89 | 11.9 (8.1) | 8.9 (15.1-43.5) | |
|  |  | hybrid diesel/electric | 310 | 17.2 (9.9) | 14.9 (22.8-56.4) | |
|  | Ottawa | 1983-2003 diesel | 392 | 17.5 (19.0) | 11.2 (20.1-156.5) | |
|  |  | 2004-2006 diesel | 241 | 15.9 (15.6) | 12.3 (18.4-125.6) | |
|  |  | 2007- diesel | 586 | 24.8 (23.6) | 18.1 (32.0-275.8) | |
|  |  | hybrid diesel/electric | 218 | 9.0 (6.8) | 6.9 (11.6-38.7) | |
|  | Vancouver | 1983-2003 diesel | 386 | 21.7 (24.6) | 12.8 (30.3-286.5) | |
|  |  | 2004-2006 diesel | 54 | 27.1 (29.0) | 18.5 (27.2-146.3) | |
|  |  | 2007- diesel | 350 | 14.6 (17.1) | 9.1 (18.1-148.1) | |
|  |  | hybrid diesel/electric | 213 | 11.6 (13.1) | 7.1 (13.2-94.7) | |
|  |  | electric* | 307 | 15.2 (12.5) | 11.6 (23.0-66.8) | |
| BC | Toronto | 1983-2003 diesel | 146 | 3.7 (2.8) | 3.0 (5-17.6) | |
| (µg/m^3^) |  | 2004-2006 diesel | 232 | 2.7 (2.8) | 1.7 (2.9-18.7) | |
|  |  | 2007- diesel | 102 | 1.2 (0.8) | 1.0 (1.6-4.2) | |
|  |  | hybrid diesel/electric | 322 | 3.3 (4.2) | 1.8 (3.7-34.7) | |
|  | Ottawa | 1983-2003 diesel | 398 | 4.1 (3.4) | 3.1 (5.2-22.5) | |
|  |  | 2004-2006 diesel | 275 | 3.6 (3.5) | 2.4 (4.6-22.8) | |
|  |  | 2007- diesel | 581 | 2.0 (2.1) | 1.2 (2.5-20.6) | |
|  |  | hybrid diesel/electric | 243 | 1.1 (0.9) | 0.8 (1.3-6.5) | |
|  | Vancouver | 1983-2003 diesel | 388 | 3.3 (2.1) | 2.8 (4.0-14.9) | |
|  |  | 2004-2006 diesel | 56 | 3.9 (5.2) | 2.6 (4.0-33.5) | |
|  |  | 2007- diesel | 308 | 2.5 (1.7) | 1.9 (3.2-10.2) | |
|  |  | hybrid diesel/electric | 183 | 2.1 (2.1) | 1.6 (2.5-18.8) | |
|  |  | electric* | 269 | 2.1 (1.3) | 1.8 (2.6-11.6) | |
| *electric buses were unique to Vancouver; ^†^unscaled DustTrak data | | | | | |  |

| **Table S6. Waiting exposures of PM_2.5_, UFP, and BC by city and bus stop type.** | | | | | |
| --- | --- | --- | --- | --- | --- |
| Pollutant | City | bus stop type | n | mean(SD) | median(p25-p75) |
| PM_2.5_^†^ | Toronto | bus stop | 623 | 25.1 (19.3) | 18.3 (35.9-128.4) |
| (µg/m^3^) |  | At grade bus station | 114 | 29.8 (17.6) | 26.5 (38.6-84.4) |
|  |  | Enclosed bus station | 101 | 38.4 (22.7) | 32.4 (57.5-110) |
|  | Ottawa | bus stop | 592 | 24.1 (18.8) | 19.1 (32.0-114.9) |
|  |  | At grade bus station | 768 | 25.0 (21.8) | 17.7 (33.2-186.6) |
|  |  | Enclosed bus station | 50 | 26.7 (21.2) | 18.6 (36.1-107.8) |
|  | Vancouver* | bus stop | 1362 | 15.0 (9.4) | 13.1 (19.1-83.4) |
|  |  | At grade bus station | 38 | 13.2 (6.7) | 12.6 (16.6-32.9) |
|  |  | Enclosed bus station | 0 | - | - |
| Ultrafine | Toronto | bus stop | 478 | 27.1 (17.5) | 23.8 (34.4-151.4) |
| Particles |  | At grade bus station | 85 | 17.5 (10.8) | 14.3 (22.1-47.7) |
| (10^3^/cm^3^) |  | Enclosed bus station | 91 | 23.0 (14.4) | 18.3 (30.2-73.5) |
|  | Ottawa | bus stop | 555 | 26.0 (37.6) | 15.3 (28.6-272.5) |
|  |  | At grade bus station | 731 | 34.0 (29.6) | 23.6 (47.6-192.1) |
|  |  | Enclosed bus station | 47 | 22.6 (15.4) | 20.0 (27.8-87.4) |
|  | Vancouver* | bus stop | 1314 | 19.4 (21.9) | 10.7 (27.0-200.0) |
|  |  | At grade bus station | 37 | 17.5 (16.1) | 11.4 (26.8-61.3) |
|  |  | Enclosed bus station | 0 | - | - |
| BC | Toronto | bus stop | 504 | 3.6 (8.2) | 1.4 (3.1-75.8) |
| (µg/m^3^) |  | At grade bus station | 102 | 3.8 (3.5) | 2.8 (4.5-17.3) |
|  |  | Enclosed bus station | 87 | 3.0 (3.7) | 1.9 (3.1-22.4) |
|  | Ottawa | bus stop | 576 | 2.4 (4.2) | 1.0 (2.3-43.5) |
|  |  | At grade bus station | 757 | 2.8 (3.5) | 1.7 (3.5-44.3) |
|  |  | Enclosed bus station | 51 | 3.7 (4.3) | 1.5 (6.9-18.3) |
|  | Vancouver* | bus stop | 1177 | 2.0 (2.2) | 1.4 (2.5-27.5) |
|  |  | At grade bus station | 35 | 1.8 (1.9) | 1.1 (2.8-8.7) |
|  |  | Enclosed bus station | 0 | - | - |
| *no enclosed bus stations were encountered during sampling in Vancouver; ^†^unscaled DustTrak data | | | | | |

**Figure S12. Composition of integrated 30-hour bus PM_2.5_ and PM_10_ samples by city.**


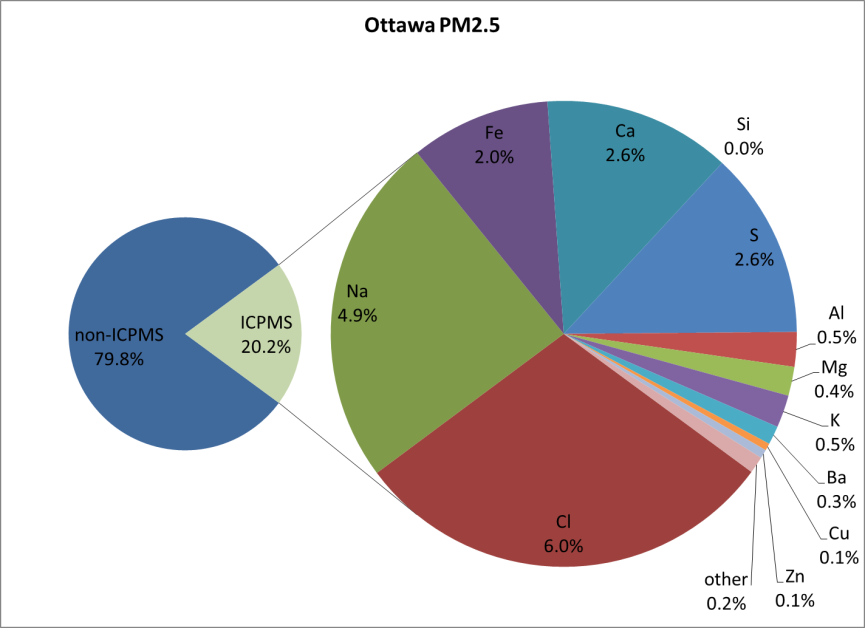

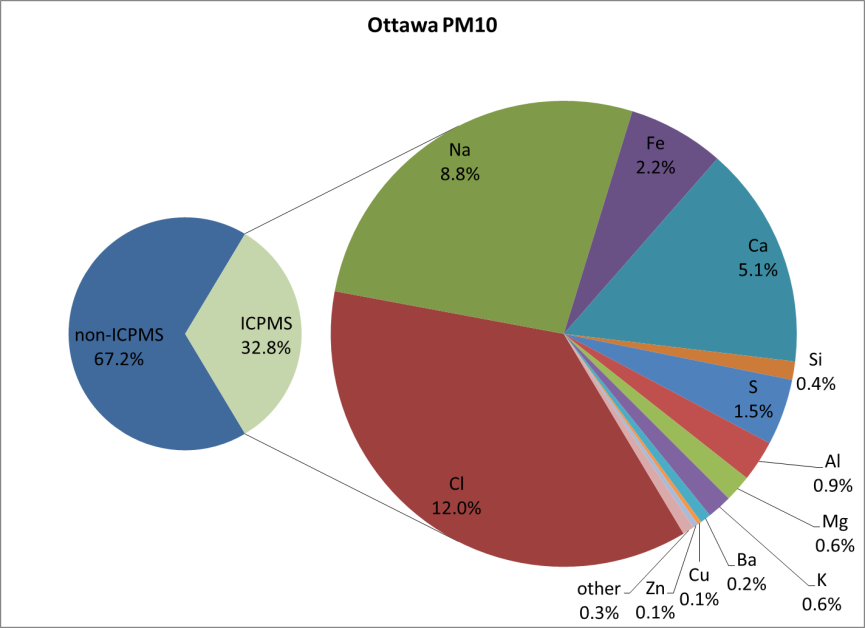

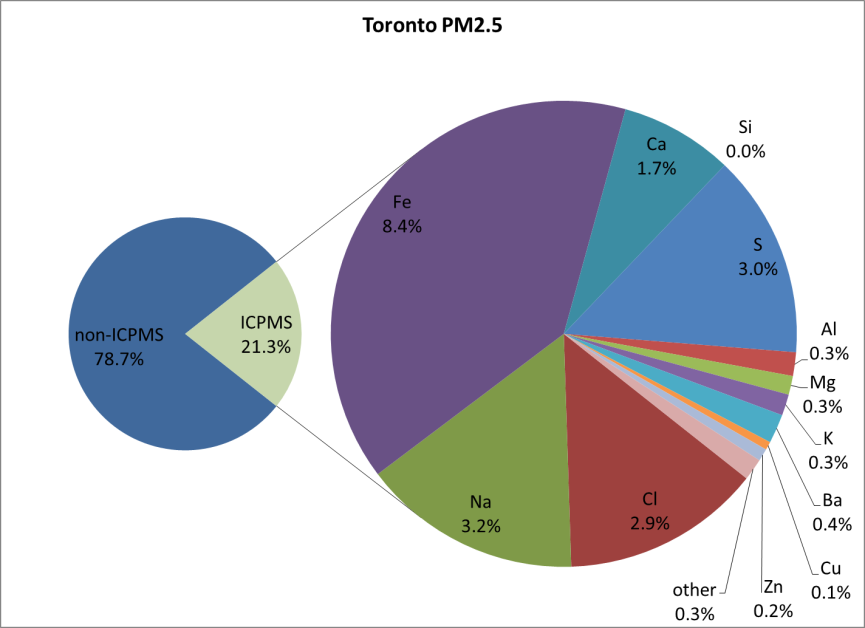

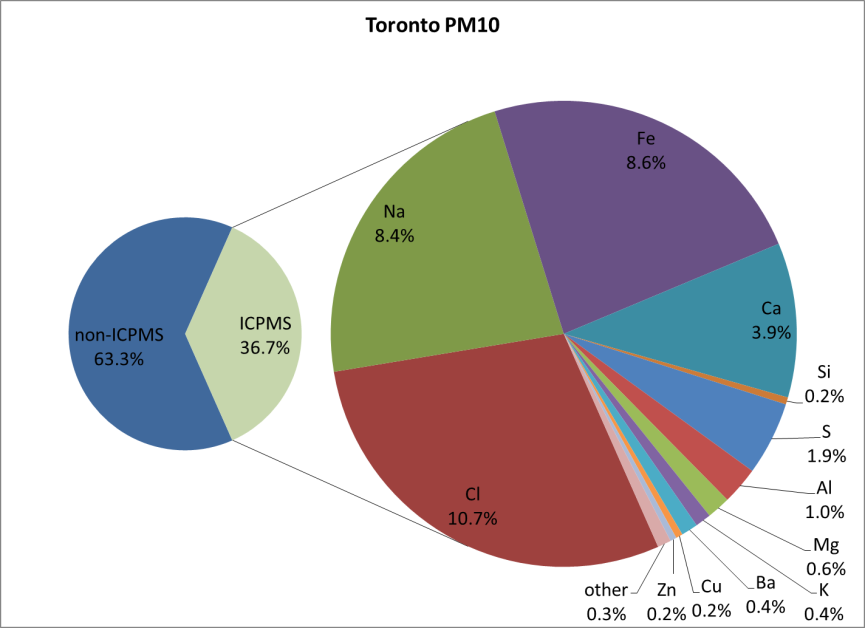

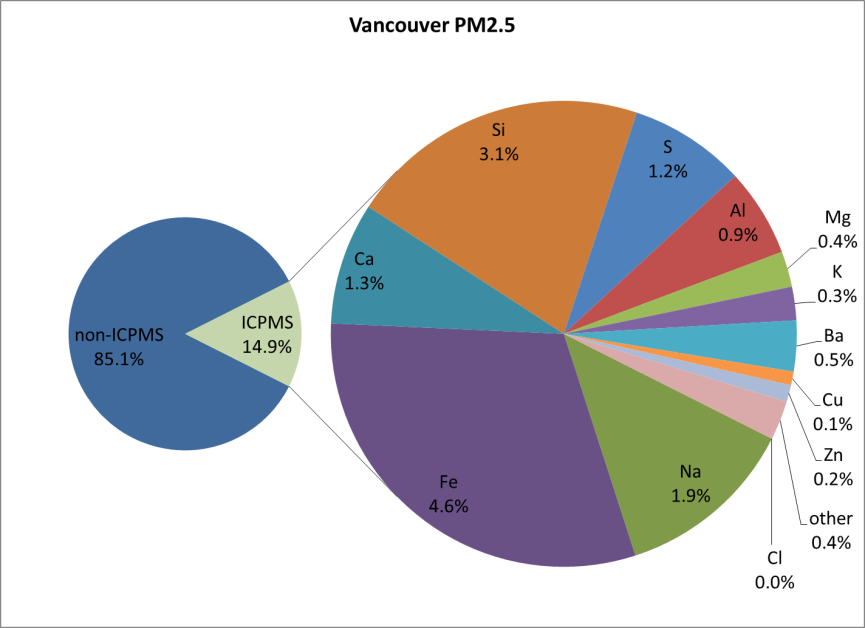

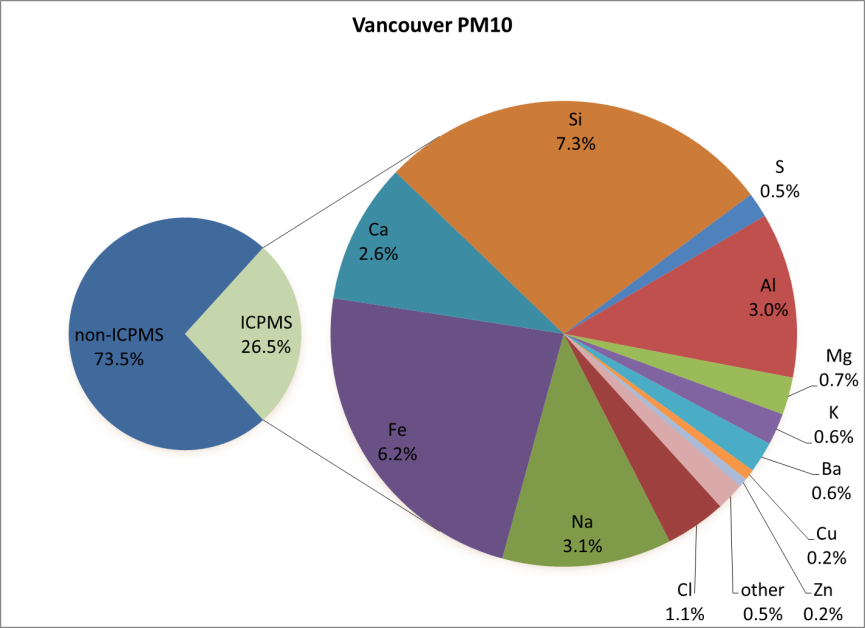


| **Table S7A. PM_2.5_ and PM_10_ and elemental composition for the Toronto bus system.** | | | | | | | | |
| --- | --- | --- | --- | --- | --- | --- | --- | --- |
| element* | PM_2.5_ (n=18) | | | | PM_10_ (n=18) | | | |
|  | %BDL | p50 | p25-p75 | %mass | %BDL | p50 | p25-p75 | %mass |
| PM | 0 | 17.6 | 16.4-24.9 | - | 0 | 58.8 | 40.8-74.8 | - |
| Ag | 0 | 0.04 | 0.03-0.05 | 0.00 | 6 | 0.09 | 0.05-0.12 | 0.00 |
| Al | 0 | 61 | 44-92 | 0.34 | 0 | 519 | 353-633 | 0.44 |
| As | 0 | 0.5 | 0.4-0.8 | 0.00 | 0 | 1 | 0.8-1.3 | 0.00 |
| B | 0 | 12.8 | 8.8-16 | 0.07 | 0 | 37.2 | 26.8-40.9 | 0.08 |
| Ba | 0 | 51 | 32-109 | 0.38 | 0 | 172 | 92-313 | 0.52 |
| Be | 11 | 0 | 0-0 | 0.00 | 6 | 0 | 0-0 | 0.00 |
| Bi | 0 | 0.1 | 0.1-0.2 | 0.00 | 0 | 0.4 | 0.3-0.6 | 0.00 |
| Ca | 0 | 347 | 199-427 | 1.60 | 0 | 2472 | 1585-3089 | 2.03 |
| Cd | 0 | 0.1 | 0.1-0.2 | 0.00 | 0 | 0.2 | 0.1-0.2 | 0.00 |
| Cl | 0 | 206 | 85-1065 | 2.76 | 0 | 2872 | 353-12634 | 5.07 |
| Co | 0 | 0.1 | 0.1-0.1 | 0.00 | 0 | 0.5 | 0.3-0.6 | 0.00 |
| Cr | 11 | 2.4 | 1.4-4.3 | 0.02 | 0 | 12.2 | 5.7-16 | 0.02 |
| Cu | 0 | 16 | 11.8-29.7 | 0.11 | 0 | 64 | 54.6-112.8 | 0.14 |
| Fe | 0 | 997 | 627-1609 | 7.85 | 0 | 4157 | 2033-4968 | 7.66 |
| Hg | 67 | 0 | 0-0.03 | 0.00 | 56 | 0.02 | 0-0.03 | 0.00 |
| K | 0 | 56 | 46-73 | 0.29 | 0 | 221 | 190-271 | 0.35 |
| Li | 0 | 0.1 | 0.1-0.1 | 0.00 | 0 | 0.4 | 0.2-0.4 | 0.00 |
| Mg | 0 | 55 | 33-76 | 0.27 | 0 | 339 | 237-491 | 0.36 |
| Mn | 0 | 13.5 | 7-16 | 0.08 | 0 | 43.1 | 26.8-55.5 | 0.08 |
| Mo | 0 | 0.7 | 0.6-1.1 | 0.01 | 0 | 2.1 | 1.6-3.8 | 0.01 |
| Na | 0 | 319 | 48-918 | 2.94 | 0 | 2699 | 211-10118 | 4.37 |
| Ni | 6 | 0.9 | 0.7-1.6 | 0.01 | 0 | 3.9 | 2.8-4.6 | 0.01 |
| P | 0 | 6.1 | 3.9-6.7 | 0.03 | 0 | 19.8 | 16.2-26.6 | 0.03 |
| Pb | 0 | 4.1 | 2.8-8.5 | 0.03 | 0 | 11.2 | 7.5-23 | 0.04 |
| S | 0 | 566 | 449-630 | 2.77 | 0 | 985 | 932-1234 | 3.00 |
| Sb | 0 | 1 | 0.7-1.2 | 0.01 | 0 | 3.3 | 2.3-4.5 | 0.01 |
| Se | 0 | 0.5 | 0.3-0.7 | 0.00 | 0 | 0.7 | 0.6-0.9 | 0.00 |
| Si | 89 | 0 | 0-0 | 0.00 | 50 | 228 | 0-1180 | 0.00 |
| Sn | 0 | 1.2 | 1-1.6 | 0.01 | 0 | 3 | 2.5-3.7 | 0.01 |
| Sr | 0 | 2.1 | 1.2-2.6 | 0.01 | 0 | 10.6 | 5.8-13.3 | 0.01 |
| Th | 0 | 0 | 0-0 | 0.00 | 0 | 0 | 0-0.1 | 0.00 |
| Ti | 0 | 6 | 4.6-8.4 | 0.04 | 0 | 29 | 23.2-35 | 0.04 |
| Tl | 0 | 0 | 0-0 | 0.00 | 0 | 0 | 0-0 | 0.00 |
| U | 0 | 0 | 0-0 | 0.00 | 0 | 0 | 0-0 | 0.00 |
| V | 0 | 0.3 | 0.3-0.4 | 0.00 | 0 | 1.3 | 1-1.6 | 0.00 |
| Zn | 0 | 29 | 18-33 | 0.19 | 0 | 74 | 57-86 | 0.16 |
| *PM mass levels are in µg/m^3,^ elemental concentrations are in ng/m^3^ | | | | | | | |  |

| **Table S7B. PM_2.5_ and PM_10_ and elemental composition for the Ottawa bus system.** | | | | | | | | |
| --- | --- | --- | --- | --- | --- | --- | --- | --- |
| element* | PM_2.5_ (n=18) | | | | PM_10_ (n=18) | | | |
|  | %BDL | p50 | p25-p75 | %mass | %BDL | p50 | p25-p75 | %mass |
| PM | 0 | 25 | 16-32 | - | 0 | 73 | 47-96 | - |
| Ag | 6 | 0.05 | 0.04-0.12 | 0.00 | 0 | 0.11 | 0.05-0.18 | 0.00 |
| Al | 0 | 88 | 52-149 | 0.67 | 0 | 526 | 411-784 | 0.45 |
| As | 0 | 0.4 | 0.3-0.6 | 0.00 | 0 | 0.6 | 0.5-1 | 0.00 |
| B | 0 | 18.1 | 10.9-21.9 | 0.05 | 0 | 35.6 | 27.6-54 | 0.07 |
| Ba | 0 | 58 | 22-99 | 0.20 | 0 | 178 | 86-235 | 0.30 |
| Be | 0 | 0.005 | 0.004-0.008 | 0.00 | 11 | 0.016 | 0.008-0.024 | 0.00 |
| Bi | 0 | 0.1 | 0-0.3 | 0.00 | 0 | 0.4 | 0.3-0.7 | 0.00 |
| Ca | 0 | 537 | 291-1128 | 3.00 | 0 | 3400 | 2199-5817 | 3.42 |
| Cd | 0 | 0.2 | 0.1-0.3 | 0.00 | 0 | 0.2 | 0.1-0.3 | 0.00 |
| Cl | 0 | 687 | 134-2303 | 4.94 | 0 | 4773 | 458-23280 | 6.99 |
| Co | 0 | 0.1 | 0.1-0.1 | 0.00 | 0 | 0.4 | 0.2-0.4 | 0.00 |
| Cr | 50 | 1.3 | 0.2-2.8 | 0.01 | 0 | 6.3 | 4.3-10.4 | 0.01 |
| Cu | 0 | 21.3 | 17.1-29 | 0.07 | 0 | 64.1 | 53.6-67.6 | 0.09 |
| Fe | 0 | 325 | 247-743 | 1.60 | 0 | 1285 | 1017-1889 | 2.25 |
| Hg | 83 | 0.002 | 0-0.008 | 0.00 | 33 | 0.043 | 0-0.078 | 0.00 |
| K | 0 | 103 | 65-156 | 0.47 | 0 | 384 | 254-548 | 0.47 |
| Li | 0 | 0.1 | 0.1-0.1 | 0.00 | 0 | 0.5 | 0.3-0.6 | 0.00 |
| Mg | 0 | 82 | 46-155 | 0.49 | 0 | 386 | 271-557 | 0.47 |
| Mn | 0 | 5.5 | 3.2-6.5 | 0.02 | 0 | 17.3 | 12.1-22.5 | 0.02 |
| Mo | 0 | 0.6 | 0.5-0.8 | 0.00 | 0 | 1.4 | 1.2-2 | 0.00 |
| Na | 0 | 931 | 80-1719 | 4.18 | 0 | 3773 | 314-16517 | 5.22 |
| Ni | 6 | 1.1 | 0.7-1.5 | 0.01 | 0 | 3.1 | 2.5-4.4 | 0.00 |
| P | 0 | 8.5 | 6.6-13.9 | 0.06 | 0 | 34.4 | 26-43.3 | 0.04 |
| Pb | 0 | 2.2 | 1.9-3.8 | 0.01 | 0 | 5.7 | 4.8-9.2 | 0.01 |
| S | 0 | 489 | 408-866 | 1.97 | 0 | 892 | 811-1234 | 2.63 |
| Sb | 0 | 0.8 | 0.5-1 | 0.00 | 0 | 2.6 | 2.1-2.8 | 0.00 |
| Se | 0 | 0.3 | 0.2-0.6 | 0.00 | 0 | 0.5 | 0.5-0.7 | 0.00 |
| Si | 61 | 0 | 0-326 | 0.00 | 33 | 421 | 0-951 | 0.99 |
| Sn | 0 | 0.8 | 0.6-1 | 0.00 | 0 | 1.9 | 1.7-2.6 | 0.00 |
| Sr | 0 | 2.8 | 1.4-4.3 | 0.01 | 0 | 11.6 | 6.9-20.7 | 0.01 |
| Th | 0 | 0.014 | 0.009-0.021 | 0.00 | 0 | 0.068 | 0.055-0.102 | 0.00 |
| Ti | 0 | 8 | 5.1-10.8 | 0.04 | 0 | 32.9 | 30-51.6 | 0.03 |
| Tl | 0 | 0.007 | 0.005-0.009 | 0.00 | 0 | 0.011 | 0.011-0.019 | 0.00 |
| U | 0 | 0.006 | 0.004-0.008 | 0.00 | 0 | 0.024 | 0.02-0.037 | 0.00 |
| V | 0 | 0.4 | 0.3-0.6 | 0.00 | 0 | 1.4 | 1-1.5 | 0.00 |
| Zn | 0 | 28 | 18-34 | 0.13 | 0 | 76 | 59-92 | 0.10 |
| *PM mass concentrations are in µg/m^3,^ elemental concentrations are in ng/m^3^ | | | | | | | |  |

| **Table S7C. PM_2.5_ and PM_10_ and elemental composition for the Vancouver bus system.** | | | | | | | | |
| --- | --- | --- | --- | --- | --- | --- | --- | --- |
| element* | PM_2.5_ (n=17) | | | | PM_10_ (n=18) | | | |
|  | %BDL | p50 | p25-p75 | %mass | %BDL | p50 | p25-p75 | %mass |
| PM | 0 | 11.2 | 10.2 - 12.9 | - | 0 | 40.2 | 36.7 - 46.6 | - |
| Ag | 0 | 0.04 | 0.02 - 0.09 | 0.00 | 0 | 0.2 | 0.14 - 0.24 | 0.00 |
| Al | 0 | 111 | 89 - 121 | 0.90 | 0 | 1308 | 984 - 1518 | 1.00 |
| As | 0 | 0.6 | 0.5 - 0.7 | 0.01 | 0 | 1 | 0.8 - 1.1 | 0.01 |
| B | 0 | 5.9 | 3.3 - 6.9 | 0.05 | 0 | 13.5 | 8.1 - 17.6 | 0.06 |
| Ba | 0 | 37 | 25 - 93 | 0.53 | 0 | 200 | 112 - 331 | 0.78 |
| Be | 0 | 0.004 | 0.004 - 0.006 | 0.00 | 0 | 0.015 | 0.013 - 0.022 | 0.00 |
| Bi | 0 | 0.1 | 0.1 - 0.1 | 0.00 | 0 | 0.8 | 0.5 - 1 | 0.00 |
| Ca | 0 | 146 | 126 - 160 | 1.23 | 0 | 1097 | 880 - 1213 | 1.32 |
| Cd | 0 | 0.1 | 0 - 0.1 | 0.00 | 0 | 0.1 | 0.1 - 0.1 | 0.00 |
| Cl | 76 | 0 | 0 - 37 | 0.08 | 0 | 297 | 210 - 620 | 0.31 |
| Co | 0 | 0.1 | 0.1 - 0.2 | 0.00 | 0 | 0.6 | 0.5 - 0.6 | 0.00 |
| Cr | 0 | 1.1 | 0.7 - 1.5 | 0.01 | 0 | 8.4 | 7.8 - 10.2 | 0.01 |
| Cu | 0 | 15.6 | 14.2 - 17.4 | 0.14 | 0 | 80.1 | 66.3 - 94.8 | 0.14 |
| Fe | 0 | 407 | 342 - 723 | 4.51 | 0 | 2415 | 1988 - 2821 | 6.00 |
| Hg | 0 | 0.01 | 0.01 - 0.01 | 0.00 | 0 | 0.03 | 0.02 - 0.04 | 0.00 |
| K | 0 | 38 | 31 - 42 | 0.34 | 0 | 236 | 195 - 285 | 0.35 |
| Li | 0 | 0.1 | 0.1 - 0.2 | 0.00 | 0 | 0.5 | 0.3 - 0.6 | 0.00 |
| Mg | 0 | 41 | 34 - 53 | 0.36 | 0 | 278 | 244 - 304 | 0.44 |
| Mn | 0 | 6.5 | 5.6 - 9.4 | 0.07 | 0 | 30.9 | 26.9 - 34 | 0.08 |
| Mo | 0 | 1.1 | 0.9 - 1.3 | 0.01 | 0 | 5.5 | 2.2 - 5.9 | 0.01 |
| Na | 0 | 205 | 183 - 238 | 1.82 | 0 | 1182 | 1080 - 1487 | 1.98 |
| Ni | 0 | 1.7 | 1.4 - 2.6 | 0.02 | 0 | 5.8 | 4.6 - 6.3 | 0.02 |
| P | 0 | 5.1 | 1.3 - 11.1 | 0.05 | 0 | 30.6 | 24.3 - 38.8 | 0.09 |
| Pb | 0 | 3.4 | 2.4 - 4.4 | 0.03 | 0 | 6.9 | 5.3 - 9.4 | 0.04 |
| S | 65 | 77 | 0 - 286 | 1.31 | 72 | 0 | 0 - 257 | 2.37 |
| Sb | 0 | 2.6 | 2 - 3.4 | 0.02 | 0 | 15.4 | 7.5 - 18.8 | 0.03 |
| Se | 0 | 0.3 | 0.3 - 0.3 | 0.00 | 0 | 0.3 | 0.3 - 0.4 | 0.00 |
| Si | 0 | 343 | 211 - 409 | 3.23 | 0 | 3182 | 2578 - 3370 | 3.39 |
| Sn | 0 | 1.5 | 1.2 - 1.9 | 0.01 | 0 | 5.9 | 4.6 - 7.7 | 0.02 |
| Sr | 0 | 1.4 | 1.1 - 1.8 | 0.01 | 0 | 8.4 | 7.8 - 10.8 | 0.02 |
| Th | 0 | 0.01 | 0 - 0.01 | 0.00 | 0 | 0.06 | 0.05 - 0.07 | 0.00 |
| Ti | 0 | 8 | 7.1 - 8.9 | 0.07 | 0 | 69.8 | 61.8 - 74.7 | 0.07 |
| Tl | 0 | 0.007 | 0.003 - 0.01 | 0.00 | 0 | 0.016 | 0.01 - 0.024 | 0.00 |
| U | 0 | 0.003 | 0.003 - 0.004 | 0.00 | 0 | 0.031 | 0.025 - 0.032 | 0.00 |
| V | 0 | 2.7 | 2.2 - 3.7 | 0.02 | 0 | 4.8 | 4 - 6 | 0.03 |
| Zn | 0 | 19 | 18 - 26 | 0.17 | 0 | 66 | 61 - 75 | 0.21 |
| *PM mass levels are in µg/m^3^, elemental concentrations are in ng/m^3^ | | | | | | | |  |

| **Table S8A. Estimates of the contribution of bus commute to overall daily exposure of PM_2.5_ and its elemental constituents in Toronto, Canada.** | | | | | | |
| --- | --- | --- | --- | --- | --- | --- |
| element | | Bus PM_2.5_ n = 18 | | Ambient PM_2.5_ n = 21 | | mean (SD)  % exposure from bus commute** |
|  |  | %  detected | mean (SD) (ng/m^3^)* | %  detected | mean (SD) (ng/m^3^)* |  |
|  | PM_2.5_ | 100 | 21.0 (9.3) | 100 | 14.2 (9.9) | 6 (2) |
|  | Al | 100 | 72.0 (39.9) | 100 | 8.7 (6.5) | 26 (11) |
|  | As | 100 | 0.63 (0.22) | 100 | 0.4 (0.3) | 7 (3) |
|  | Ba | 100 | 80.1 (67.6) | 100 | 1.8 (1.0) | 59 (15) |
|  | Cd | 100 | 0.16 (0.18) | 100 | 0.08 (0.05) | 8 (7) |
|  | Cr | 89 | 4.8 (6.7) | 81 | 0.4 (0.2) | 27 (21) |
|  | Cu | 100 | 23.7 (19.6) | 100 | 2.1 (1.0) | 30 (15) |
|  | Fe | 100 | 1648 (1977) | 100 | 20.6 (21.1) | 70 (19) |
|  | Mn | 100 | 15.9 (14.0) | 100 | 1.6 (1.2) | 27 (14) |
|  | Mo | 100 | 1.16 (1.18) | 95 | 0.2 (0.2) | 17 (12) |
|  | Ni | 94 | 1.26 (0.98) | 86 | 0.3 (0.2) | 15 (11) |
|  | Pb | 100 | 5.4 (3.2) | 100 | 1.7 (1.1) | 12 (6) |
|  | Sb | 100 | 1.1 (0.6) | 100 | 0.4 (0.3) | 12 (4) |
|  | Se | 100 | 0.51 (0.21) | 81 | 0.5 (0.5) | 4 (2) |
|  | Sn | 100 | 1.2 (0.4) | 100 | 0.3 (0.1) | 17 (4) |
|  | Sr | 100 | 2.1 (1.1) | 100 | 0.4 (0.2) | 20 (9) |
|  | Ti | 100 | 8.4 (8.5) | 67 | 0.5 (0.2) | 39 (14) |
|  | V | 100 | 0.35 (0.12) | 100 | 0.3 (0.2) | 6 (2) |
|  | Zn | 100 | 39.6 (56.7) | 100 | 13.6 (6.7) | 10 (9) |

***PM_2.5_ levels are in µg/m^3^**

****Percent contribution of 66 minute bus commute to overall daily exposure (eq 1).**

| **Table S8B. Estimates of the contribution of bus commute to overall daily exposure of PM_2.5_ and its elemental constituents in Ottawa, Canada.** | | | | | | |
| --- | --- | --- | --- | --- | --- | --- |
| element | | Bus PM_2.5_ n = 18 | | Ambient PM_2.5_ n = 21 | | mean (SD)  % exposure from bus commute** |
|  |  | %  detected | mean (SD) (ng/m^3^)* | %  detected | %  detected |  |
|  | PM_2.5_ | 100 | 33.0 (30.9) | 100 | 8.9 (6.6) | 13 (10) |
|  | Al | 100 | 220 (374) | 100 | 11.8 (20.5) | 32 (23) |
|  | As | 100 | 0.47 (0.23) | 100 | 0.5 (0.7) | 5 (3) |
|  | Ba | 100 | 65.3 (46.6) | 100 | 1.3 (0.8) | 60 (20) |
|  | Cd | 100 | 0.25 (0.29) | 100 | 0.08 (0.08) | 10 (9) |
|  | Cr | 50 | 2.3 (3.0) | 62 | 0.3 (0.1) | 19 (18) |
|  | Cu | 100 | 24.1 (9.7) | 91 | 1.7 (1.1) | 37 (9) |
|  | Fe | 100 | 526 (437) | 100 | 9.4 (6.5) | 64 (15) |
|  | Mn | 100 | 6.7 (6.0) | 100 | 0.9 (0.7) | 23 (13) |
|  | Mo | 100 | 0.66 (0.23) | 52 | 0.09 (0.08) | 24 (7) |
|  | Ni | 94 | 1.95 (2.44) | 62 | 0.2 (0.1) | 22 (16) |
|  | Pb | 100 | 3.1 (1.9) | 100 | 1.2 (0.8) | 10 (5) |
|  | Sb | 100 | 1.05 (0.91) | 95 | 0.4 (0.5) | 10 (6) |
|  | Sn | 100 | 0.91 (0.49) | 95 | 0.2 (0.1) | 19 (8) |
|  | Sr | 100 | 4.0 (3.4) | 100 | 0.4 (0.3) | 26 (15) |
|  | Ti | 100 | 13.8 (16.6) | 67 | 0.6 (1.1) | 41 (19) |
|  | V | 100 | 0.53 (0.42) | 86 | 0.2 (0.3) | 8 (5) |
|  | Zn | 100 | 41.9 (50.7) | 100 | 12 (11.4) | 12 (11) |

***PM_2.5_ levels are in µg/m^3^**

****Percent contribution of 66 minute bus commute to overall daily exposure (eq 1).**

| **Table S8C. Estimates of the contribution of bus commute to overall daily exposure of PM_2.5_ and its elemental constituents in Vancouver, Canada.** | | | | | | |
| --- | --- | --- | --- | --- | --- | --- |
| element | | Bus PM_2.5_ n = 17 | | Ambient PM_2.5_ n = 21 | | mean (SD)  % exposure from bus commute** |
|  |  | %  detected | mean (SD) (ng/m^3^)* | %  detected | %  detected |  |
|  | PM_2.5_ | 100 | 12.0 (2.9) | 100 | 4.8 (2.2) | 11 (4) |
|  | Al | 100 | 108 (37) | 95 | 8.8 (6.9) | 38 (15) |
|  | As | 100 | 0.63 (0.22) | 100 | 0.3 (0.2) | 8 (2) |
|  | Ba | 100 | 63.7 (49.4) | 100 | 1.5 (0.9) | 57 (18) |
|  | Co | 100 | 0.14 (0.05) | 52 | 0.02 (0.02) | 21 (9) |
|  | Cu | 100 | 16.4 (4.3) | 81 | 1.5 (0.7) | 32 (7) |
|  | Fe | 100 | 543 (262) | 95 | 9.1 (6.4) | 70 (15) |
|  | Mn | 100 | 8.0 (3.0) | 100 | 1.7 (1.5) | 18 (9) |
|  | Mo | 100 | 1.1 (0.3) | 81 | 0.2 (0.1) | 22 (6) |
|  | Ni | 100 | 2.7 (3.1) | 100 | 0.7 (0.5) | 14 (9) |
|  | Pb | 100 | 3.69 (1.96) | 95 | 1.00 (1.02) | 18 (13) |
|  | Sb | 100 | 2.74 (1.22) | 100 | 0.2 (0.1) | 36 (10) |
|  | Sr | 100 | 1.59 (0.71) | 100 | 0.2 (0.1) | 24 (9) |
|  | V | 100 | 2.92 (1.31) | 100 | 0.9 (0.7) | 13 (6) |
|  | Zn | 100 | 20.3 (6.2) | 100 | 10.3 (7.3) | 8 (2) |

***PM_2.5_ levels are in µg/m^3^**

****Percent contribution of 66 minute bus commute to overall daily exposure (eq 1).**

# 3. References

1. Wallace, L. A.; Wheeler, A. J.; Kearney, J.; Van Ryswyk, K.; You, H.; Kulka, R. H.; Rasmussen, P. E.; Brook, J. R.; Xu, X. Validation of continuous particle monitors for personal, indoor, and outdoor exposures. J. Expos. Sci. Environ. Epidemiol. **2011**, 21, 49-64.

2. Van Ryswyk, K.; Anastasopolos, A. T.; Evans, G.; Sun, L.; Sabaliauskas, K.; Kulka, R.; Wallace, L.; Weichenthal, S. Metro Commuter Exposures to Particulate Air Pollution and PM2. 5-Associated Elements in Three Canadian Cities: The Urban Transportation Exposure Study. Environmental Science \& Technology **2017**, 51, 5713-5720.
